# TRPM8 levels determine tumor vulnerability to channel agonists

**DOI:** 10.1002/1878-0261.70049

**Published:** 2025-05-22

**Authors:** Alessandro Alaimo, Francesco Giuseppe Carbone, Kristi Buzo, Nicole Annesi, Sacha Genovesi, Annalisa Lorenzato, Karen Widmann, Michela Libergoli, Elisa Marmocchi, Giovanni Bertalot, Alberto Brolese, Mauro Giulio Papotti, Luca Molinaro, Orazio Caffo, Mattia Barbareschi, Alberto Bardelli, Alessandro Romanel, Sabrina Arena, Andrea Lunardi

**Affiliations:** ^1^ Department of Cellular, Computational and Integrative Biology (CIBIO), University of Trento Italy; ^2^ Surgical Pathology Santa Chiara Hospital‐APSS Trento Italy; ^3^ Department of Oncology University of Torino Torino Italy; ^4^ Candiolo Cancer Institute, FPO–IRCCS Candiolo (TO) Italy; ^5^ Centre for Medical Sciences‐CISMed, University of Trento Italy; ^6^ Department of General Surgery & HPB Unit Santa Chiara Hospital‐APSS Trento Italy; ^7^ Department of Pathology University of Torino and AOU Città della Salute e della Scienza di Torino Italy; ^8^ Medical Oncology Santa Chiara Hospital‐APSS Trento Italy; ^9^ IFOM ETS – The AIRC Institute of Molecular Oncology Milan Italy

**Keywords:** breast cancer, colorectal cancer, D‐3263, ion channel, lung cancer, prostate cancer, TRPM8

## Abstract

Targeted therapies have pervasively enhanced clinical protocols and significantly improved survival and quality of life of cancer patients. Mostly grounded on small molecules and antibodies targeting deregulated mechanisms in cancer cells, precision oncology approaches are limited to a few tumor types because of the paucity of clinically actionable targets. Here, we report a comparative analysis of the cation channel transient receptor potential melastatin 8 (TRPM8; also known as transient receptor potential cation channel subfamily M member 8) in lung, breast, colorectal, and prostate cancers. Our findings reveal high levels of channel expression in cores of all four carcinomas, irrespective of reduced expression of its RNA. Importantly, cancer cell lines that represent the various tumor types consistently show that sub‐lethal chemotherapy dosages combined with the TRPM8 agonist D‐3263 have a synergistic lethal effect. In addition, administration of D‐3263 increases the cytotoxicity of 5‐FU/Oxaliplatin in patient‐derived colorectal cancer organoids, depending on the levels of TRPM8. Overall, our study strengthens the candidacy of TRPM8 as a molecular target for precision oncology approaches and paves the way for the design of basket trials for its clinical testing in TRPM8‐high tumors.

## Graphical abstract

TRPM8 is a Ca^2+^ permissive channel. Regardless of the amount of its transcript, high levels of TRPM8 protein mark different tumors, including prostate, breast, colorectal, and lung carcinomas. Targeting TRPM8 with channel agonists stimulates inward calcium currents followed by emptying of cytosolic Ca^2+^ stores in cancer cells. TRPM8‐induced calcium cytotoxicity transforms ineffective chemotherapy into a lethal treatment for cancer cells.

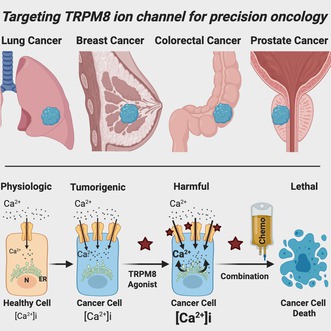

**Abbreviations**
ATCC: American Type Culture Collection
BC: breast cancer
BME: basement membrane extract
BPE: bovine pituitary extract
BSA: bovine serum albumin
CRC: Colorectal cancer
DAB: 3,3′‐Diaminobenzidine
ECL: enhanced chemiluminescence
EGF: epidermal growth factor
FBS: fetal bovine serum
FFPE: formalin‐fixed paraffin‐embedded
FITC: fluorescein isothiocyanate
GAPDH: glyceraldehyde‐3‐phosphate dehydrogenase
IR: ionizing radiation
KSFM: keratinocyte serum‐free medium
NSCLC: non‐small cell lung cancer
PARP: poly (ADP‐ribose) polymerase
PCa: prostate cancer
PDO: patient‐derived organoid
PDXO: patient‐derived xenoorganoid
PFA: paraformaldehyde
PI: propidium iodide
PVDF: polyvinylidene difluoride
RNAseq: RNA sequencing
RPLP: ribosomal protein lateral stalk subunit P
RTK: receptor tyrosine kinase
siRNA: short interfering RNA
TBS: tris‐buffered saline
TCGA: The Cancer Genome Atlas
TMA: tissue microarray
TRPM2: transient receptor potential melastatin 2
TRPM8: transient receptor potential melastatin 8
5‐FU: 5‐fluorouracil

## 1 Introduction

Omics investigation through cutting‐edge technologies has proven crucial to unwind the molecular heterogeneity and the complexity of cancer biology [1, 2]. Ever more frequently, defined molecular signatures are used in the clinic to classify solid and liquid tumors in actionable categories and stratify patients on precise oncological protocols [3, 4]. Targeted therapeutic interventions directed against specific oncogenic mechanisms or cancer cell vulnerabilities have significantly changed the prognosis of lethal tumors such as lung, breast, prostate, and colorectal cancers (https://www.cancer.gov/about‐cancer/treatment/types/targeted‐therapies/approved‐drug‐list). Inhibition of receptor tyrosine kinases (RTKs), enzymes involved in DNA repair, and components of the immune checkpoints are among the privileged strategies adopted by modern oncology to counteract cancer progression. Generally effective for precise classes of molecularly stratified tumors, mechanisms of resistance almost invariably arise, and the disease inevitably relapses [5]. To overcome therapy resistance, a large portfolio of more potent and selective drugs is under continuous clinical investigation (https://clinicaltrials.gov/), while, on the other hand, a tireless effort of pre‐clinical research feeds the list with novel promising druggable candidates.

In this frantic search for better treatments and novel therapeutic strategies, pharmacological gating of ion channels represents an inestimable resource for oncology that deserves great attention [6].

The *Transient Receptor Potential* ‐*TRP*‐ genes encode for cell membrane ion channels highly conserved from yeast to mammals with critical roles in sensory perception and cellular physiology [7]. In mammals, the TRP family is composed of multi‐gene subfamilies including TRPA1 (ankyrin), TRPCs (canonical), TRPMLs (mucolipin), TRPMs (melastatin), TRPPs (polycystin), and TRPVs (vanilloid) [8]. Most TRPs are non‐selective cation channels whose mechanisms of gating range from variations in transmembrane potential or temperature to binding of specific ligands. By depolarizing the cell membrane when activated, some TRPs function as intracellular Ca^2+^ release channels, thus having a crucial role in cell biology. From a clinical perspective, mutations in *TRP* genes have been implicated in hereditary disorders (TRP channelopathies) such as skeletal dysplasia, neurodegenerative syndromes, kidney dysfunctions, and pain [7, 9, 10]. Because of their location on the cell surface and the presence of a specific ligand binding pocket, several TRP channels are archetypal drug targets whose pharmacological gating could have relevant clinical implications ranging from pain relief to respiratory diseases, from neurological and psychiatric disorders to diabetes and cancer [7, 11]. Among the members of the TRP family, *Transient Receptor Potential cation channel subfamily M member 8* ‐*TRPM8*‐ gene is reported in literature as abundantly expressed in the luminal compartment of normal prostate epithelium [12, 13, 14, 15]. Although its role remains poorly defined, TRPM8 rises in prostate cancer (PCa) compared to normal adjacent tissue at both RNA and protein levels, suggesting pro‐tumorigenic activities of the channel. Notably, a growing number of publications highlight a keen sensitivity of different pre‐clinical models of PCa to the pharmacologic tuning of TRPM8 [16, 17, 18]. Our previous works [12, 13, 14] demonstrate a massive apoptotic response of aggressive cross‐species cellular models of PCa to a combination of sub‐lethal standard‐of‐care treatments (IR, chemo‐, hormone‐therapy) with potent TRPM8 agonists (WS‐12 and D‐3263).

Here, we describe the efficacy of TRPM8 targeting in three other major human killers such as lung, breast, and colorectal cancers. Despite the low amount of TRPM8 RNA reported by The Cancer Genome Atlas (TCGA) for tumors other than PCa, immunolocalization of TRPM8 identified breast, lung, and colorectal cancer cores on a multi‐tumor microarray (TMA) with levels of the channel comparable to PCa. Importantly, the combination of sub‐lethal doses of standard chemotherapy with the potent TRPM8 agonist D‐3263 induces a pervasive apoptotic program in breast, lung, colon, and prostate cancer cell lines. Knock‐down of TRPM8 prevents cell death in all cell lines, thus proving D‐3263 specificity. Similarly, D‐3263 increases 5‐FU/Oxaliplatin cytotoxicity in patient‐derived colorectal cancer organoids, depending on TRPM8 levels of protein expression.

Overall, these results shed light on the value of TRPM8 as a molecular target for the treatment of distinct tumor types, regardless of tissue of origin and RNA amount, but selected based on high levels of channel expression.

## 2 Materials and methods

### 2.1 Cell culture

LNCaP (#CRL‐1740; RRID:CVCL_0395), PC‐3 (#CRL‐1435; RRID:CVCL_0035), VCaP (#CRL‐2876; RRID:CVCL_2235), RWPE‐1 (#CRL‐11609; RRID:CVCL_3791), A549 (#CCL‐185; RRID:CVCL_0023), HCT116 (#CCL‐247; RRID:CVCL_0291), MCF7 (#HTB‐22; RRID:CVCL_0031), SK‐MEL5 (#HTB‐70; RRID:CVCL_0527), G361 (#CRL‐1424; RRID:CVCL_1220), and A375 (#CRL‐1619; RRID:CVCL_0132) cell lines were purchased from the American Type Culture Collection (ATCC, LGC Standards). The cells were grown in RPMI medium (Sigma, St. Louis, MO, USA) or in DMEM medium (MCF7, VCaP, A375, G361, SK‐MEL5; Invitrogen, ThermoFisher Sci, Waltham, MA, USA) either supplemented with 10% fetal bovine serum (FBS; Sigma), 100 U·mL^−1^ penicillin, and 100 μg·mL^−1^ streptomycin (Pen/Strep; Invitrogen and 2 mm L‐Glutamine (Invitrogen)). RWPE‐1 cells were cultured in KSFM medium (Invitrogen) supplemented with 0.05 mg·mL^−1^ bovine pituitary extract (BPE), 5 ng·mL^−1^ EGF, and 1% Pen/Strep. All cells were cultured in a humidified incubator at 37 °C and 5% CO_2_ and were passaged in conformity with the manufacturer's protocols. Cell lines were routinely tested for Mycoplasma (MycoAlert Mycoplasma Detection Kit, Lonza) and authenticated for specific markers by western blot and RT‐qPCR.

### 2.2 Human samples

A high‐density tissue microarray (TMA) of colon, rectum, breast, lung, and prostate tumors, containing 208 cases/208 cores (192 cases of tumor and 16 cases of normal tissues) was purchased from US Biomax, Inc. (MC2081a). Eight colorectal cancer TMAs containing 80 cases (160 cores, 1 core of tumor tissue and 1 core of control normal mucosa from each patient) were generated from FFPE stored samples at the Operative Unit of Anatomy Pathology of the Santa Chiara Hospital (Trento, Italy) upon study approval of the local Ethical Committee of the Santa Chiara Hospital (Trento, Italy) (Prot.:1946 I.D.:112786962). Samples were collected randomly with regard to stage and grade. Human colorectal cancer and prostate specimens were derived from segmental resections of the large bowel at the Santa Chiara Hospital of Trento (August–November 2011) and radical prostatectomy at the Molinette Hospital of Turin (Italy) (January–February 2020), respectively. Prostate cancer patients were enrolled with written informed consent on a study protocol approved by the Ethical Committee of the Molinette Hospital, Turin (Rep. Int. 0009136). Frozen colorectal tumor samples collected with patients' written informed consent were recovered from the tissue bank of the Santa Chiara Hospital (Trento, Italy). Study methodologies conforming to the standards set by the Declaration of Helsinki were approved by the local ethics committees of Molinette Hospital in Turin, Italy, and Santa Chiara Hospital in Trento, Italy, respectively.

### 2.3 RNA isolation and quantitative PCR

RNA extraction was performed using the RNAeasy Micro Kit (Qiagen) following the manufacturer's instructions. The concentration and quality of the RNA were evaluated by NanoDropTM 2000c spectrophotometer (ThermoFisher Sci, Waltham, MA, USA) and agarose electrophoresis. Total RNA (1 μg) was reverse transcribed into cDNA using iScript™ cDNA synthesis Kit (Biorad) according to the manufacturer's protocol. Quantitative Real‐time PCR was carried out on a CFX96 qPCR Thermal cycler (Biorad) using KAPA SYBR® FAST qPCR Master Mix (Kapa biosystems, Wilmington, MA, USA). The data were normalized to the housekeeping genes *Glyceraldehyde‐3‐phosphate dehydrogenase* (*GAPDH*) or *beta‐ACTIN* (*b‐ACTIN*) transcripts for the analysis of TRPM8 expression in cancer cell lines or to the geometrical mean of *GAPDH*, *RPLP*, and *18S* transcripts for the analysis of TRPM8 expression in human prostate and colorectal samples, analyzed as relative RNA levels of the cycle threshold (Ct) value, then converted to fold change. PCR analyses were performed with at least *n* = 2 independent biological replicates. Specific sense and antisense PCR primers used in the study were: *TRPM8*, GATTTTCACCAATGACCGCCG (Fw), CCCCAGCAGCATTGATGTCG (Rv); *GAPDH*, AGCCACATCGCTCAGACACC (Fw), GTACTCAGCGCCAGCATCG (Rv); *RPLP*, CGTCCTCGTGGAAGTGACAT (Fw), TAGTTGGACTTCCAGGTCGC (Rv); *18S*, CAGCCACCCGAGATTGACA (Fw), TAGTAGCGACGGGCGGTGTG (Fw); *bACTIN*, AGAGATGGCCACGGCTGCTT (Fw), ATTTGCGGTGGACGATGGAG (Rv).

### 2.4 Western blot

Immunoblotting was performed as previously reported [12, 14]. Briefly, equal amounts of proteins were separated by SDS/PAGE, transferred onto a PVDF membrane (AmershamTM Hybond™; Fisher Scientific, Buckinghamshire, UK) and blocked with 5% BSA or 5% non‐fat dry milk in 1× TBS‐Tween. The following primary antibodies were used: anti‐TRPM8 (ACC‐049, Alomone Labs and ab3243; Abcam), ‐TRPM2 (PA5‐102844, ThermoFisher Science), ‐PARP (9542; Cell Signaling Technology, Danvers, MA, USA), ‐Cleaved PARP (Asp214, 5625, Cell Signaling Technology), ‐Cleaved Caspase‐3 (Asp175, 9661, Cell Signaling Technology) and ‐β‐Actin (A2228, Sigma). The reaction was revealed by using ECL Select WB Detection Reagent (GE Healthcare, Little Chalfont, UK) with an Alliance LD2 system and software (UVITEC). Immunoblots were performed in three independent biological replicates and quantified with ImageJ (v2.0.0‐rc‐69/1.52i); representative data are shown.

### 2.5 Small interfering RNA silencing

Cells were plated on a six‐well plate (2 × 10^5^ cells/well) and transiently transfected at about 60% confluence with targeting siRNAs against human TRPM8 or TRPM2 (100 nm) or negative control siRNA (see below) using Lipofectamine® LTX Reagent (Life Tech, ThermoFisher Sci, Waltham, MA, USA) and OptiMEM media (Invitrogen) as described in the manufacturer's protocol. For TRPM8, the following siRNA sequences were used: siRNA1 GGUGCUUUGGAUUCUCACGG (Ambion 104 796, Life Tech), siRNA2 GGAUGCCCUGACAUCUUUCU (Ambion 104 798, Life Tech), siCtr Silencer® Negative Control #1 (Ambion AM4611, Life Tech). The sequences of TRPM2 siRNA (si‐TRPM2#1, si‐TRPM2#, si‐NC) were obtained from [19] and purchased from Eurofins Genomics.

### 2.6 Drugs

Docetaxel (01885, 10 mm), Oxaliplatin (09512, 50 mm) and 5‐Fluorouracil (F6627, 500 mm), and WS‐12 (W0519, 10 mm) were purchased from Sigma; D‐3263 (D‐195, 10 mm) was obtained from Alomone Labs and was resuspended in dimethyl sulfoxide (DMSO) to achieve the indicated stock concentrations. All drugs were maintained as stock solutions and stored at −80 °C or −20 °C. In each experiment, the same volume of solvent used for tested drugs and chemicals was added to the control solution.

### 2.7 FACS analysis

Cells were cultured at about 60% confluence in six‐well dishes and treated for 24 h as indicated in the figures. Cell death and apoptotic rates were determined with Annexin‐V‐FITC and propidium iodide (PI) staining according to the manufacturer's instructions (Annexin‐V FITC Kit; Miltenyi Biotec, Bergisch Gladbach, Germany). For FACS analysis, a BD FACSymphony™ A1 Cell Analyzer (BD Biosciences, Franklin Lakes, NJ, USA) was used, and data were analyzed with flowjo software (Treestar, Ashland, USA).

### 2.8 Crystal violet cell cytotoxicity assay

Six‐well plates with 70% confluent cells were treated as indicated in the figures. Twenty‐four hours later, cells were washed with PBS, fixed with 10% formalin (Sigma), washed again with PBS, and stained with 0.1% Crystal Violet (Sigma) solution (in 20% methanol) for 30 min. Afterward, cells were washed with dH20, dried, and Crystal Violet was extracted with 10% acetic acid for 30 min. For quantification, absorbance was measured at 595 nm. The experiments were performed in triplicates, and images were taken with a Chemidoc XRSF (Biorad).

### 2.9 Immunohistochemistry

Cells were grown on coverslips, fixed with 4% PFA, incubated with peroxidase inhibitor solution, saturated for 1 h at RT and, finally, incubated with primary antibodies (anti‐TRPM8 Alomone Labs ACC‐049 or Abcam Ab3243) O/N at 4 °C. Coverslips were washed and then incubated with biotin‐conjugated secondary antibody (Jackson ImmunoResearch, West Grove, PA, USA) for 1 h at RT, washed again, and incubated for 1 h at RT with Avidin‐Biotin complex (Vectastain® Elite ABC Peroxidase kit, Vector Labs, PK‐6100, Burlington, CA, USA) according to the manufacturer's instructions. Samples were incubated with DAB revelation solution and counterstained with hematoxylin before mounting the coverslips. TMAs were subjected to immunohistochemical analyses carried out at the Department of Histopathology (S. Chiara Hospital, Trento, Italy) using an automatic immunostainer (BOND‐III platform, Leica Biosystems, Wetzlar, Germany). Antigen retrieval was carried out with optimized BOND reagents (Bond epitope retrieval solution 1, Leica Biosystems) at pH 6 for 20 min. BOND compact polymer detection solution (Leica Biosystems) was used for the detection, as previously described [12, 13, 14]. The primary antibodies used to detect TRPM8 were the Alomone Labs ACC‐049 or Abcam Ab3243 diluted at 1:800 for use on the BOND system. Samples histology and TRPM8 immunostaining were independently reviewed by three pathologists (M.B., F.G.C., and G.B.) to ensure appropriate assignment of the following scores: absence of staining (0), weak (1), moderate (2), and high (3) signal intensity.

### 2.10 Colorectal organoids

The PDOs and patient‐derived xenoorganoids (PDXOs) were established and maintained in the culture as described in full details in [20]. Briefly, tumor samples were obtained from patients enrolled at Niguarda Cancer Center (Milan, Italy) (patient #2–5) and from University of Rostock (Germany) (patient#1) in a timeframe between 2006 and 2019. All patients provided informed written consent, samples were procured, and the study was conducted in accordance with the Declaration of Helsinki and under the approval of the local Independent Ethical Committee (protocol 194/2010), Italian Ministry of the Health and the Ethics Committee of the Medical faculty of the University of Rostock, in accordance with generally accepted guidelines for the use of human material. Patient #1 and patient #3 organoids were initially established in the laboratory of Prof. Bardelli from PDX models (PDXOs) as fully described in [20]. Patient #2, patient #4, and patient #5 organoids were established directly from tissue biopsy obtained at the time of surgery. Organoids from patient#2 were established at INGM (Istituto Nazionale Genetica Molecolare “Romeo ed Enrica Invernizzi”, Milan, Italy), whereas organoids from patients #4 and #5 were established at Candiolo Cancer Institute. Organoids were processed and treated following the protocol previously described in [20, 21]. Briefly, organoids were seeded as single cell at a density of 4000 to 6000 cells per well in 96‐well plates precoated with basement membrane extract (BME; Cultrex BME Type 2; Amsbio, Cambridge, MA, USA). Treatment was performed 4 days after seeding, once grown organoids were visible. Indicated concentrations of drugs, D‐3263 (1.5 μm) and 5‐Fluorouracil (0.5 μm) + Oxaliplatin (1 μm) were added automatically by Tecan D300e Digital Dispenser in fresh 150 μL medium containing 2% BME. A total of 4 μmol/L MG‐132 was used as a positive control; DMSO served as a negative control. The viability was assayed at the end of the experiment after 5 days of treatment by CellTiter‐Glo Luminescent Cell Viability assay (Promega, Madison, WI, USA) with modifications (full details in [21]). The results derive from two independent biological experiments, each with six technical replicates.

### 2.11 Statistics

Data are expressed as mean ± sd of three biological replicates, unless otherwise indicated. Statistical analyses were carried out using GraphPad Prism 8.0, with the threshold of significance set at <0.05.

## 3 Results

### 3.1 TRPM8 immunostaining reveals underestimated channel expression in human lung, breast, and colorectal carcinomas

**Fig. 1:**
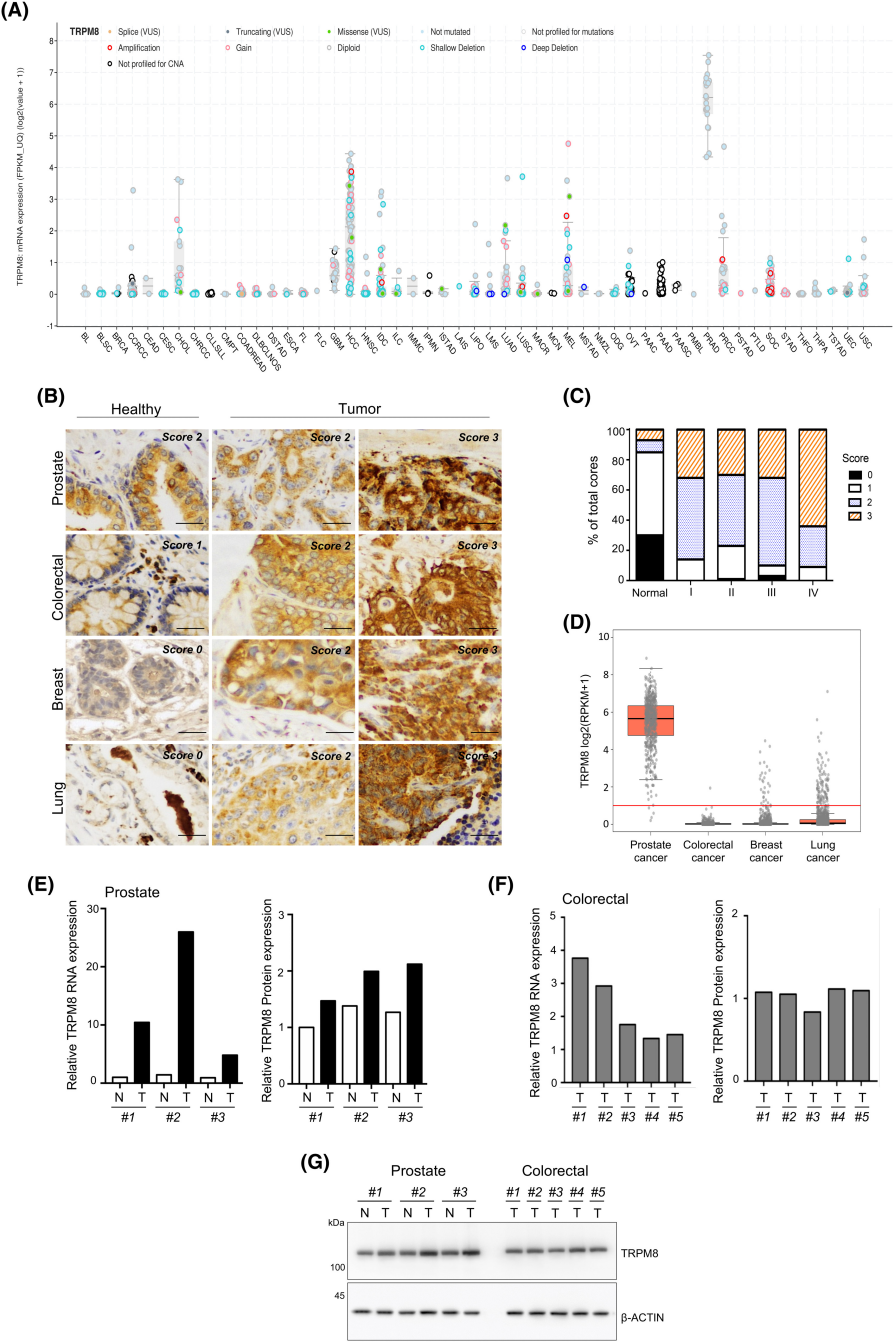
TRPM8 RNA and protein expression in solid cancers. (A) Landscape of TRPM8 transcript levels in primary tumor samples across different tissues (horizontal axis) was retrieved from The Cancer Genome Atlas (TCGA) datasets of cBioPortal. Panel display boxplots illustrating data distributions; boxes represent the median and interquartile range, while whiskers indicate variability beyond the quartiles, with individual data points shown above the plots. (B) Representative images of TRPM8 immunolocalization in healthy and malignant prostate, colorectal, breast, and lung tissue samples spotted on a commercial tissue microarray (TMA). TRPM8 immunostaining was scored as absent (0), weak (1), moderate (2) or high (3) (scale bar 20 μm). (C) TRPM8 score related to tumor stage (*n* = 48 cores per cancer type; *n* = 4 cores per normal tissue type). (D) Direct comparison of TRPM8 RNA expression across prostate, colorectal, breast, and lung cancers. Panel display boxplots illustrating data distributions; boxes represent the median and interquartile range, while whiskers indicate variability beyond the quartiles, with individual data points shown above the plots. (E, F) Quantification of TRPM8 RNA expression and protein amount in matched normal prostate tissue (N) and prostate cancer (T) samples isolated from *n* = 3 radical prostatectomies of prostate cancer (PCa) patients (E), and *n* = 5 independent colorectal cancer (CRC) samples (F). (G) Western blot of TRPM8 protein in the samples described in (E, F).

Transcriptional profiling of the TRPM8 gene based on the Cancer Genome Atlas RNAseq datasets defines prostate tissue and, even more, prostate carcinoma as the primary sites for TRPM8 expression (Fig. 1A) [12]. Hepatocellular carcinoma follows, while all other tumors show TRPM8 RNA levels close to the detection threshold in almost the totality of samples (Fig. 1A) [12]. Experimental evidence accrued over the past years by our group has frequently pointed out a sharp dichotomy between the levels of TRPM8 transcript and the amount of the protein in prostate cell lines and human samples [12, 14], raising doubts about the predictability of TRPM8 channel expression based on its RNA levels. To analyze the status of the TRPM8 channel in a group of solid tumors other than prostate cancer, a Tissue Microarray was purchased from US Biomax, Inc. (MC2081a) and stained with a validated antibody against TRPM8 (Alomone #ACC‐049; [12, 13, 14]) at the Anatomic Pathology Operative Unit of the Santa Chiara Hospital of Trento. Blind analyses by three experienced pathologists (MB, FGC, GB) defined TRPM8 channel expressed at very low levels in normal lung, breast, and intestinal epithelium with a marked increase in corresponding tumors frequently associated with the tumor stage (Fig. 1B,C). Notably, comparative analysis of lung, breast, colorectal, and prostate carcinoma cores spotted on the same TMA defined high levels of TRPM8 protein in different cores of all four tumor types analyzed (Fig. 1B; Tables S1, S2), regardless of the relative expression of *TRPM8* RNA across them (Fig. 1D). To further investigate the TRPM8 channel in solid tumors other than prostate cancer, RNA and protein amounts were studied in a set of three matched normal and tumor prostate samples collected from patients undergoing radical prostatectomy and five colorectal cancer samples collected from patients after segmental resection of the large bowel (Fig. 1E–G). As expected, both TRPM8 RNA and protein were expressed in normal prostate tissues and raised in matched neoplastic lesions (Fig. 1E,G, Fig. S1A). In sharp contrast to the RNAseq data showing almost undetectable levels of TRPM8 RNA in CRC (Fig. 1A,D), all five CRC samples were characterized by TRPM8 RNA and protein expression, as also indicated by immunohistochemistry studies (Fig. 1B). Of note, in both types of tumor, the relative amounts of TRPM8 protein between samples do not reflect the relative amount of its RNA in the same samples.

### 3.2 TRPM8 activation twists sub‐lethal chemotherapy into effective cancer treatment

**Fig. 2:**
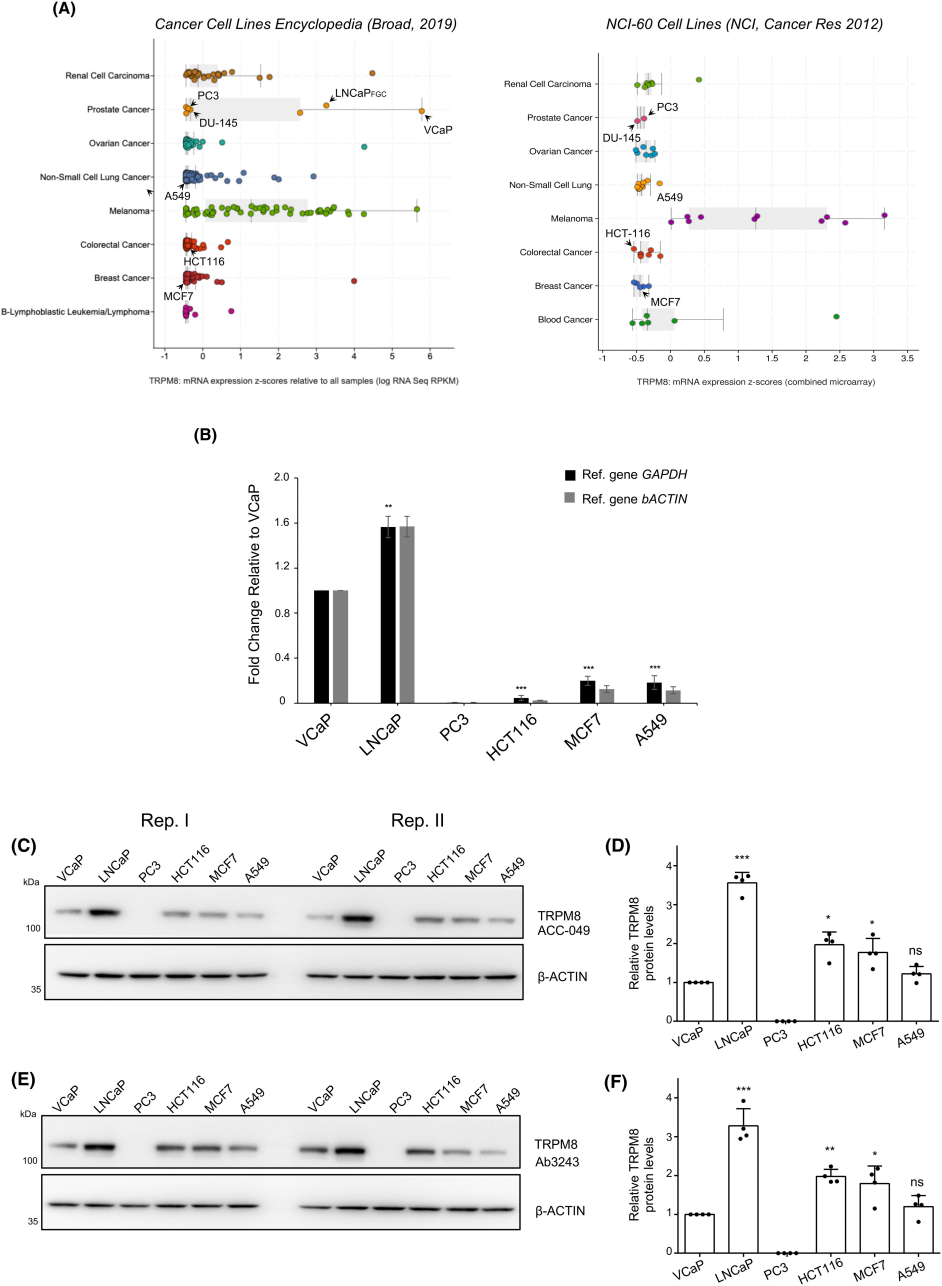
Variable amounts of TRPM8 channel in tumor cells with low levels of its coding transcript. (A) TRPM8 RNA expression in cancer cell lines described in the NCI‐60 cell lines and Cancer Cell Lines Encyclopedia projects (retrieved from cBioPortal). (B) Quantitative reverse transcription polymerase chain reaction (RT‐qPCR) comparative analysis of TRPM8 RNA expression in prostate (VCaP, LNCaP, PC3), colon (HCT116), breast (MCF7) and lung (A549) cancer cells. (C–F) Western blot replicas I and II of TRPM8 in VCaP, LNCaP, PC3, HCT116, MCF7, A549 cell lines with the Alomone ACC‐049 (C) and Abcam Ab3243 (E) antibodies, and relative quantification of TRPM8 protein (D, F) in the *n* = 4 independent replicas shown in C–E and Fig. S2C. β‐Actin is used as loading control and normalizer. Data are presented as mean ± standard deviation (sd) of three (Alomone ACC‐049 antibody) and four (Abcam Ab3243 antibody) independent experiments. ***P ≤ 0.001, **P ≤ 0.01, *P ≤ 0.05. Statistical analysis was performed using Student's *t*‐test.

Classical cell line models of colorectal cancer (CRC, HCT116), breast cancer (BC, MCF7), and non‐small cell lung cancer (NSCLC, A549) were chosen to study TRPM8 expression and cellular response to the administration of the channel agonist D‐3263, compared to widely used TRPM8‐positive (VCaP and LNCaP) and TRPM8‐negative (PC3) prostate cancer (PCa) cell lines [12]. Analysis of the NCI‐60 cell lines and Cancer Cell Line Encyclopedia datasets ([22, 23, 24]) defined the amount of TRPM8 RNA in HCT116, MCF7, and A549 comparable to that of TRPM8‐negative PC3 and DU‐145 prostate cancer cells (Fig. 2A). RT‐qPCR studies showed slightly more TRPM8 transcript in HCT116, MCF7, and A549 cells compared to PC3, but significantly less (five to ten times) compared to TRPM8‐positive VCaP and LNCaP prostate cancer cells (Fig. 2B, Fig. S1B). Regardless of the amount of RNA, HCT116, MCF7, and A549 express levels of TRPM8 protein ranging between those expressed in VCaP and LNCaP cells (2 times more than VCaP, half compared with LNCaP; Fig. 2C–F, Fig. S1C), which have been shown to be highly sensitive to the combination of sub‐lethal doses of standard cancer treatments (*e.g*., IR, HT, CT) with the potent TRPM8 agonists WS‐12 or D‐3263 [12]. Interestingly, the study of SK‐MEL5, G361, and A375 melanoma cell lines further pointed out the unpredictability of TRPM8 protein expression depending on the levels of its transcript (Fig. S1D).

**Fig. 3:**
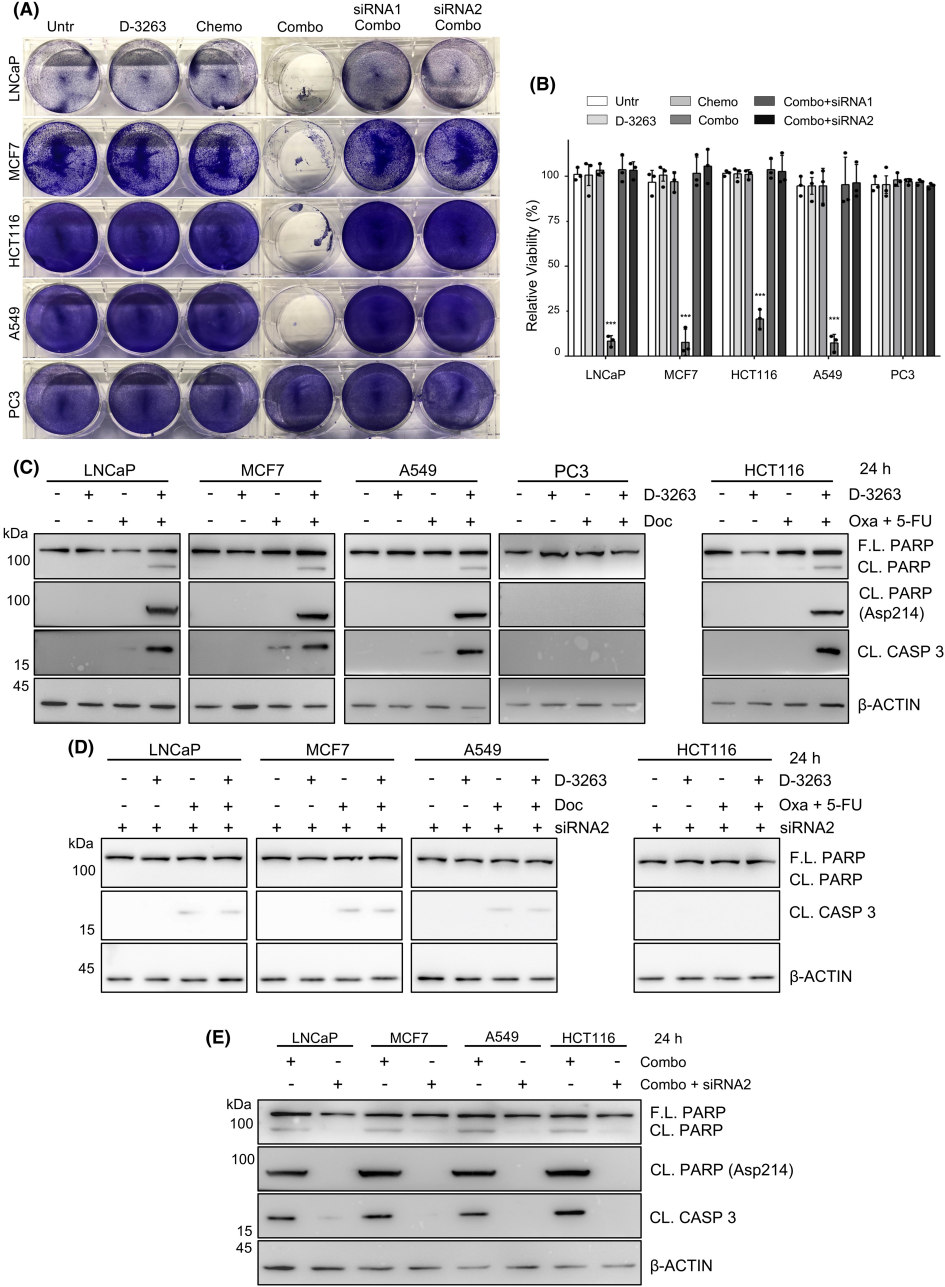
Lethal synergy between TRPM8 agonist D‐3263 and chemotherapy in cancer cells. (A) Representative crystal violet staining of LNCaP, HCT116, MCF7, A549, and PC3 cells untreated or treated with the indicated drugs for 24 h (chemotherapy = Docetaxel for LNCaP, MCF7, A549, and PC3; 5‐fluorouracil (5‐FU + Oxaliplatin for HCT116). TRPM8 knock‐down (siRNA1 and siRNA2) confirmed D‐3263 specificity. (B) Quantification of the viability of treated cells compared with untreated controls. Data are presented as mean ± standard deviation (sd) of *n* = 3 independent experiments (A, Fig. S2C). ***P < 0.001. Statistical analysis was performed using Student's *t*‐test. (C, D) Western blot analysis of Caspase 3 and Parp cleavage in LNCaP, HCT116, MCF7, A549, and PC3 cells untreated or treated with the indicated drugs for 24 h (C). TRPM8 knock‐down (siRNA2) confirmed D‐3263 specificity (D). (E) Direct comparison of D‐3263 efficacy in wild type and TRPM8 knocked down (KD) cancer cells. Western blot analysis in C–E was repeated with *n* = 2 sets of biologically independent samples.

Clinical protocols define Docetaxel as the standard genotoxic agent for advanced PCa, BC, and NSCLC, while advanced CRC is often treated with FOLFOX, a combination of 5‐FU and Oxaliplatin. To test the possible contribution of TRPM8 activation to the antitumor efficacy of selected chemotherapies, LNCaP, MCF7, and A549 cells were treated for 12 and 24 h with sub‐lethal doses of Docetaxel (10 nM) or TRPM8 agonist D‐3263 (1 μm) as single agents or with the combination of both. HCT116 cells received sub‐lethal doses of 5‐FU (10 μm)/Oxaliplatin (2 μm), D‐3263 (1 μm) or WS‐12 (1 μm) as single agents or a combination of all three. None of the TRPM8‐positive cell lines showed marks of cell death after 12 h of treatments (Fig. S2A). Twelve hours later (24 h of treatment), no signs of toxicity were found in D‐3263 or WS‐12‐treated cells; Docetaxel induced a slight cleavage only of Caspase 3 in LNCaP, MCF7, and A549, while the combination of chemotherapy and D‐3263 or WS‐12 triggered terminal apoptosis in more than 70% of the populations in all cell lines (Fig. 3A–C, Figs S2B,C, S3, S4A). TRPM8‐null PC3 cells, as well as LNCaP, MCF7, A549, and HCT116 cells with knocked down levels of the TRPM8, were refractory to the combination (Fig. 3A–E, Figs S2B–G, S3, S4A).

According to the literature, TRPM2 is the closest member of the TRPM subfamily to TRPM8. The two channels share the structure and prevalent permeability to calcium ions; moreover, based on their amino acid sequence, most of the key residues involved in agonist binding are conserved [25, 26, 27, 28]. To investigate the specificity of D‐3263 targeting of TRPM8 for cancer cell response, we profiled the expression of the TRPM2 ion channel in LNCaP, PC3, HCT116, MCF7, and A549 cancer cell lines. Western blotting showed comparable expression of TRPM2 in LNCaP, PC3, and HCT116 cell lines; A549 cells were characterized by a very low amount of TRPM2 channel, whereas the channel was not detected in MCF7 (Fig. S4B). The expression of TRPM2 in PC3 cells, which are TRPM8‐null and refractory to D‐3263, and its absence in MCF7 cells, which are TRPM8‐positive and sensitive to D‐3263, suggest the negligibility of TRPM2 for D‐3263 activity. In line with this, TRPM2 knock‐down in D‐3263‐sensitive TRPM8‐positive LNCaP and HCT116 cancer cells did not alter the ability of D‐3263 to raise the cancer cell killing activity of standard chemotherapy (Fig. S4C–F).

### 3.3 D‐3263 enhances 5‐FU/oxaliplatin toxicity in patient‐derived CRC organoids

**Fig. 4:**
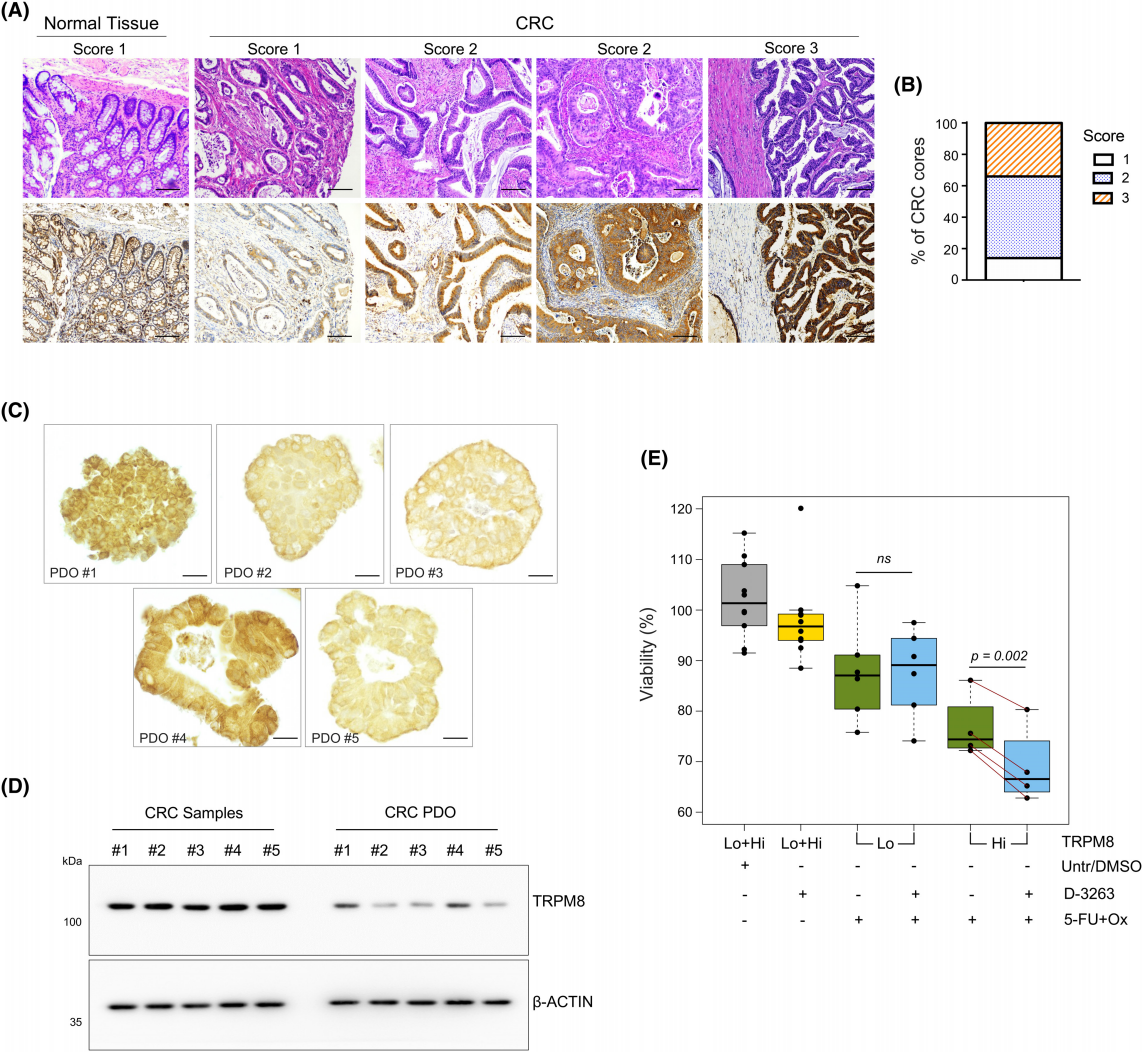
Efficacy of D‐3263 + 5‐FU + Oxaliplatin in CRC organoids raises with the amount of TRPM8. (A) TRPM8 immunolocalization in a dedicated homemade tissue microarray (TMA) of colorectal cancer (scale bar 100 μm; ACC‐049). Representative images of colorectal cancer (CRC) with different levels of TRPM8 staining and relative scores. Score 1: weak expression, score 2: moderate expression, score 3: high expression. (B) Distribution of TRPM8 immunostaining scores across the colorectal cancer (CRC) specimens (*n* = 79 independent cores). (C) Immunolocalization of TRPM8 in *n* = 5 different lines of patient‐derived colorectal cancer (CRC) organoids (scale bar 20 μm). (D) Western blot of TRPM8 from colorectal cancer (CRC) specimens (*n* = 5 independent samples) derived from the tissue bank of the Santa Chiara Hospital of Trento and—independent—*n* = 5 patient‐derived organoids (PDO). Western blot analysis in D was repeated twice. (E) Relative viability of colorectal cancer (CRC) organoids subjected to the indicated treatments or left untreated to serve as control pooled together based on TRPM8 protein amount (high (Hi): PDO #1 and PDO #4; low (Lo): PDO #2, PDO #3 and PDO #5). Distribution of viability measures across the different conditions and stratifications is shown using boxplots. Paired two‐sample *t*‐test was used to compare the viability of organoid lines. Data are presented as mean ± standard deviation (sd) of two independent biological experiments, each with six technical replicates.

Colorectal cancer is the solid tumor showing the highest divergence between TRPM8 RNA and protein expression. Although the increased amount of TRPM8 in tumors is supposed to promote cancer cell fitness through activation of Ca^2+^‐dependent pathways, we recently demonstrated a tight connection of TRPM8 RNA with anti‐cancer immunity [29]. Because of the extensive interaction of the intestinal tissue with the immune system, we decided to gain knowledge of TRPM8 in colorectal cancer. We assembled a dedicated TMA bearing 80 independent cores representing different stages of disease, each paired with a core from the corresponding adjacent normal tissue (Table S3). TMA sections were stained with two independent specific antibodies against TRPM8 (Alomone ACC‐049 and Abcam Ab3243) and analyzed by three experienced pathologists (MB, FGC, GB). The analyses confirmed the increased amount of the channel in cancer lesions compared with healthy tissue (Fig. 4A,B, Fig. S5, Table S3), with no obvious correlations between the amount of TRPM8 and cancer stage.

To simulate a possible approach of precision oncology exploiting TRPM8 targeting, five well‐characterized CRC organoid lines [20, 21] were enrolled in a pre‐clinical trial testing D‐3263 and 5‐FU/Oxaliplatin as single agents or in combination. Immunolocalization studies showed different amounts of TRPM8 across the five organoid lines (Fig. 4C). Western blotting confirmed the heterogeneity of channel expression between the organoid lines, but also underlined substantially less expression of TRPM8 in CRC organoids compared to unrelated CRC tissues derived from the tissue bank of the Santa Chiara Hospital of Trento (Fig. 4D). Similar to cell lines, D‐3263 was ineffective when administered as a single agent (Fig. 4E). Treatment with 5‐FU and Oxaliplatin slightly reduced the viability of three CRC organoid lines PDO #2, PDO #3, and PDO #5 by ~10%, while the PDO #1 and PDO #4 lines exceeded 20%. Noteworthy, D‐3263 synergized with 5‐FU/Oxaliplatin in PDO #1 and PDO #4 expressing higher amounts of TRPM8, leaving the effect of chemotherapy unaffected in the organoid lines with lower levels of the channel (Fig. 4C–E).

## 4 Discussion

Genomics and transcriptomics profiling of thousands of human cancers coupled with accurate functional studies have substantially increased our knowledge of cancer biology and significantly improved the clinical approach to different forms of tumors. However, the lack of cutting‐edge technologies allowing a deep characterization of the cancer proteome in wide cohorts of patients downsizes the discovery of the molecular mechanisms involved in tumorigenesis and, in turn, the landscape of possible strategies to defeat cancer. In the list of underestimated contributors to cancer prognosis, ion channels sit in the front row. Rarely mutated, deleted, or amplified in cancer, their transcriptional deregulation in neoplastic lesions compared to healthy tissues is generally mediocre and not enough to raise deep interest in the scientific community. This oversimplified reasoning can lead to misjudging the clinical relevance of seemingly negligible puzzles of the mosaic, which could instead represent valuable oncologic targets [12, 13, 14, 18, 30].

*TRPM8* gene expression highly characterizes the luminal compartment of normal prostate. Almost invariably, RNA levels increase in hormone‐sensitive primary tumors and metastases but then decrease significantly in castration‐resistant tumors [12, 13, 14]. The amount of TRPM8 protein parallels its transcript and rises in hormone‐sensitive primary and metastatic tumors with respect to no‐tumoral cells but, unexpectedly, it remains well‐expressed in castration‐resistant PCa [12, 13, 14]. The dichotomy between RNA levels and protein amount of TRPM8 is also evident in different prostate cell lines, thus defining protein detection a more reliable method than RNA profiling for studying TRPM8. Consistent with these observations, we demonstrate that breast, lung, and colorectal cancers exhibit variable amounts of TRPM8 regardless of the very low level of the RNA reported by the Cancer Genome Atlas (TCGA) RNAseq datasets. According to the TCGA data, RNA expression of TRPM8 in normal tissues is generally low (except for prostate tissue) with minimal variability among samples, which consistently mirrors the amount of the channel detected for IHC in healthy epithelia, thus suggesting that the RNA/TRPM8 discrepancy might be a tumor‐specific event rather than a generalized condition. In widely used colorectal, breast, and lung cancer cell lines, TRPM8 channel is functional and stimulation with the potent agonist D‐3263 drives lethal cytotoxicity when combined with sub‐lethal doses of standard chemo‐agents routinely used in the clinic for the treatment of advanced stages of disease [31]. Identification of novel therapeutic routes improving cancer prognosis implies finding treatments with an acceptable efficacy/toxicity ratio. Knock‐down experiments in all cell lines define a net correlation between levels of the channel and efficacy of D‐3263, as also previously shown for prostate cell lines treated with a different agonist of TRPM8 (WS‐12, [12]). The reduced amount of TRPM8 protein in normal tissues can thus justify the negligible toxicity described in both rats and humans treated with D‐3263 [32, 33]. Noteworthy, Dendreon Pharmaceuticals in 2009 pioneered a small interventional Phase I clinical trial (NCT00839631) enrolling cancer patients diagnosed with different types of advanced solid tumors (prostate, colon, breast, and lung cancer, among others) to test D‐3263. Side effects were limited to cold sensations, while three advanced prostate cancers showed signs of disease stabilization [33]. These findings support the relevance that TRPM8 targeting may have in oncology, particularly for the treatment of those tumors that, by immunohistochemistry, express high levels of the channel. The formal demonstration that D‐3263 works in TRAMP‐C1 and C2 mouse cell line models of PCa [14] paves the way for pre‐clinical *in vivo* trials in orthotopically transplanted immune‐competent syngeneic C57BL/6 mice. A similar characterization of Trpm8 expression and function in mouse C57BL/6 MC‐38 and BALB/c CT‐26 is ongoing and will potentially expand our *in vivo* pre‐clinical platform to colorectal cancer. On the other hand, the ability to rapidly study the amount of TRPM8 and the contribution to therapy of channel agonists in patient‐derived tumor organoids perfectly meets the principles of precision oncology based on co‐clinical strategies [34, 35, 36, 37, 38].

From a mechanistic perspective, TRPM8 activation in cells characterized by higher expression of the channel is expected to drive inward calcium currents with the consequent emptying of the intracellular Ca^2+^ stores and, finally, calcium cytotoxicity [12]. However, experiments aimed at detecting and quantifying free cytosolic Ca^2+^ through the ratiometric fluorescent dye Fura‐2 have often proved inconclusive in cancer cells treated with WS‐12 and D‐3263 [12, 14]. The calcium‐sensitive bioluminescent protein Aequorin could help carefully evaluate Ca^2+^ flux at the level of specific subcellular compartments [39, 40]. Of note, these studies did not include concomitant treatment of cancer cells with a therapy, which might instead integrate TRPM8 action and promote emptying of intracellular Ca^2+^ stores. Although calcium remains the main suspect, we cannot rule out the possibility that cytotoxicity associated with potent channel agonists may depend on the deregulation of the homeostasis of Na^2+^ and K^+^ to which TRPM8 is also permeable.

## 5 Conclusions

Overall, this work demonstrates the lethal synergy of TRPM8 agonists and standard chemotherapy in the four major killers among human cancers, shedding light on the importance that ion channels may have as molecular targets for precision oncology.

## Conflict of interest

S. Arena (SA) reports personal fees from MSD Italia and a patent (Italian patent application No. 102022000007535) outside the submitted work. A. Bardelli (ABa) declares the following competing financial interests: receipt of grants/research support from Neophore, AstraZeneca, Boehringer; receipt of honoraria or consultation fees from Guardant Health; stock shareholder: Neophore, Kither Biotech; member of the SAB of Neophore. No disclosures were reported by the other authors.

## Author contributions

AA: Conceptualization, data curation, validation, investigation, visualization, methodology, writing‐original draft; FGC: Data curation, validation, investigation, visualization, methodology; KB: Data curation, validation, investigation, methodology; NA: Data curation, formal analysis, visualization, methodology; SG: Data curation, investigation, methodology; ALo.: investigation; KW: Data curation, methodology; ML: Data curation, methodology; EM: Data curation, validation, visualization; ABr: Data curation, investigation; MGP: Data curation, investigation; LM: Data curation, investigation; OC: Data curation, investigation; GB: Data curation, investigation. MB: Data curation, investigation; ABa: investigation, funding acquisition; AR: Data curation, investigation; SA: Data curation, investigation, validation, funding acquisition, reviewing; ALu: Conceptualization, data curation, visualization, supervision, funding acquisition, writing original draft, reviewing, and editing.

## Peer review

The peer review history for this article is available at https://www.webofscience.com/api/gateway/wos/peer‐review/10.1002/1878‐0261.70049.

## Supporting information


**Fig. S1.** Comparable amounts of TRPM8 protein in prostate, colorectal, breast, and lung cancer cells. (A) Uncropped western blot relative to Fig. 1F. As previously described in Alaimo et al. (2020), lysates of LNCaP cells show both the full‐length (128 kDa, Plasma Membrane) and the shorter (35 kDa, Endoplasmic Reticulum) forms of TRPM8. (B) Amount of TRPM8 RNA in cancer cell lines relative to MCF7. Data are normalized using *GAPDH* (*upper panel*, *n* = 3) or *bACTIN* (*lower panel*, *n* = 2) expression as housekeeping genes. (C) Western blot replicas III and IV of TRPM8 in VCaP, LNCaP, PC3, HCT116, MCF7, A549 cell lines with the Alomone ACC‐049 (*upper panel*) and Abcam Ab3243 (*lower panel*) antibodies. β‐Actin is used as loading control. (D) TRPM8 RNA and protein quantification in melanoma cancer cell lines SK‐MEL5, G361, and A375 (*n* = 2).

**Fig. S2.** Activation of TRPM8 promotes chemotoxicity in cancer cells. (A) Western blot analysis of Caspase 3 and Parp cleavage in untreated or treated LNCaP, HCT116, MCF7, and A549 cell lines with the indicated drugs for 12 h. (B) Schematic representation of the experiments in C (chemotherapy = Docetaxel for LNCaP, MCF7, A549, and PC3; 5‐fluorouracile (5‐FU) + Oxaliplatin for HCT116. TRPM8 knock‐down = siTRPM8 #1 and siTRPM8 #2). (C) Crystal violet staining of LNCaP, HCT116, MCF7, A549, and PC3 cells untreated or treated for 24 h with the indicated drugs. (D, E) Western blotting of TRPM8 in LNCaP (D), HCT116, MCF7, and A549 (E) cell lines untransfected (Unt), transfected with control siRNA (siCtrl) or siRNAs targeting TRPM8 (siRNA1 and siRNA2). β‐Actin is used as loading control. Quantification is relative to the untreated (Untr) condition for each cell line. (F, G) Western blotting (F) and immunohistochemistry (G) of TRPM8 in HCT116, MCF7, and A549 cell lines transfected with control siRNA (−) or siRNA1 targeting TRPM8 (+). β‐Actin is used as loading control. Secondary antibody alone (Ab‐II^ary^) is used as negative control.

**Fig. S3.** Combination of D‐3263 with chemotherapy induces apoptosis in TRPM8 positive cancer cells. Cell death rate by fluorescence‐activated cell sorting (FACS) with Annexin‐V‐FITC and propidium iodide (PI) staining of LNCaP, HCT116, MCF7, A549, and PC3 cells untreated or treated with the indicated drugs for 24 h (chemotherapy = Docetaxel for LNCaP, MCF7, A549, and PC3; 5‐fluorouracile (5‐FU) + Oxaliplatin for HCT116. TRPM8 knock‐down = siTRPM8 #2).

**Fig. S4.** TRPM2 ion channel in cancer cell lines. (A) Western blotting analysis of TRPM8, PARP, and Caspase 3 in HCT116 cancer cell line treated with WS‐12 (1 M) and chemotherapy. (B) Western blotting analysis of TRPM2 expression in LNCaP, PC3, HCT116, MCF7 and A549 cancer cell lines. (C) Western blotting analysis showing TRPM2 knock‐down by specific siRNAs (siRNA1 and siRNA2) in LNCaP, PC3, HCT116 cancer cell lines. (D, E) Western blot analysis of Caspase 3 and PARP cleavage in LNCaP, HCT116, MCF7 cells transfected with control siRNA (siCtr) (D) or TRPM2 siRNA (siRNA1) (E) and untreated or treated with the indicated drugs for 24 h. (F) Western blotting analysis showing TRPM2 knock‐down by siRNA1 in LNCaP, PC3, HCT116 cancer cell lines treated with D‐3263 and chemotherapy in D and E.

**Fig. S5.** TRPM8 ion channel expression in colorectal cancer specimens. (A) TRPM8 in a serial section to that shown in Fig. 4A of a homemade dedicated colorectal cancer tissue microarray (scale bar 100 μm; Ab3243). Representative images of CRC with different levels of TRPM8 staining and relative scores. Score 0: no expression; score 1: weak expression, score 2: moderate expression, score 3: high expression. (B) Distribution of TRPM8 immunostaining scores in colorectal cancer (CRC) samples with Alomone ACC‐049 and Abcam AB‐3243 antibodies, showing higher detection efficiency of ACC‐049 than Ab‐3243 at the same dilution, but consistent distribution of relative scores across samples.

**Table S1.** Multi‐organ tissue microarray US Biomax, Inc. (MC2081a) and TRPM8 immunostaining score.

**Table S2.** Clinical description of multiple organ tumor tissue array.

**Table S3.** Clinical description of Colorectal Cancer tissue microarray (TMA) and TRPM8 immunostaining score.

## Data Availability

All data needed to evaluate the conclusions in the paper are presented in the paper and/or Supplementary Materials. Additional data is available upon request from the corresponding authors.
