# Supplementary figures and images for: TRPM8 levels determine tumor vulnerability to channel agonists
Source: Mol Oncol. 2025 May 22;19(10):2905–20. doi: 10.1002/1878-0261.70049 (PMC12515718; doi:10.1002/1878-0261.70049)

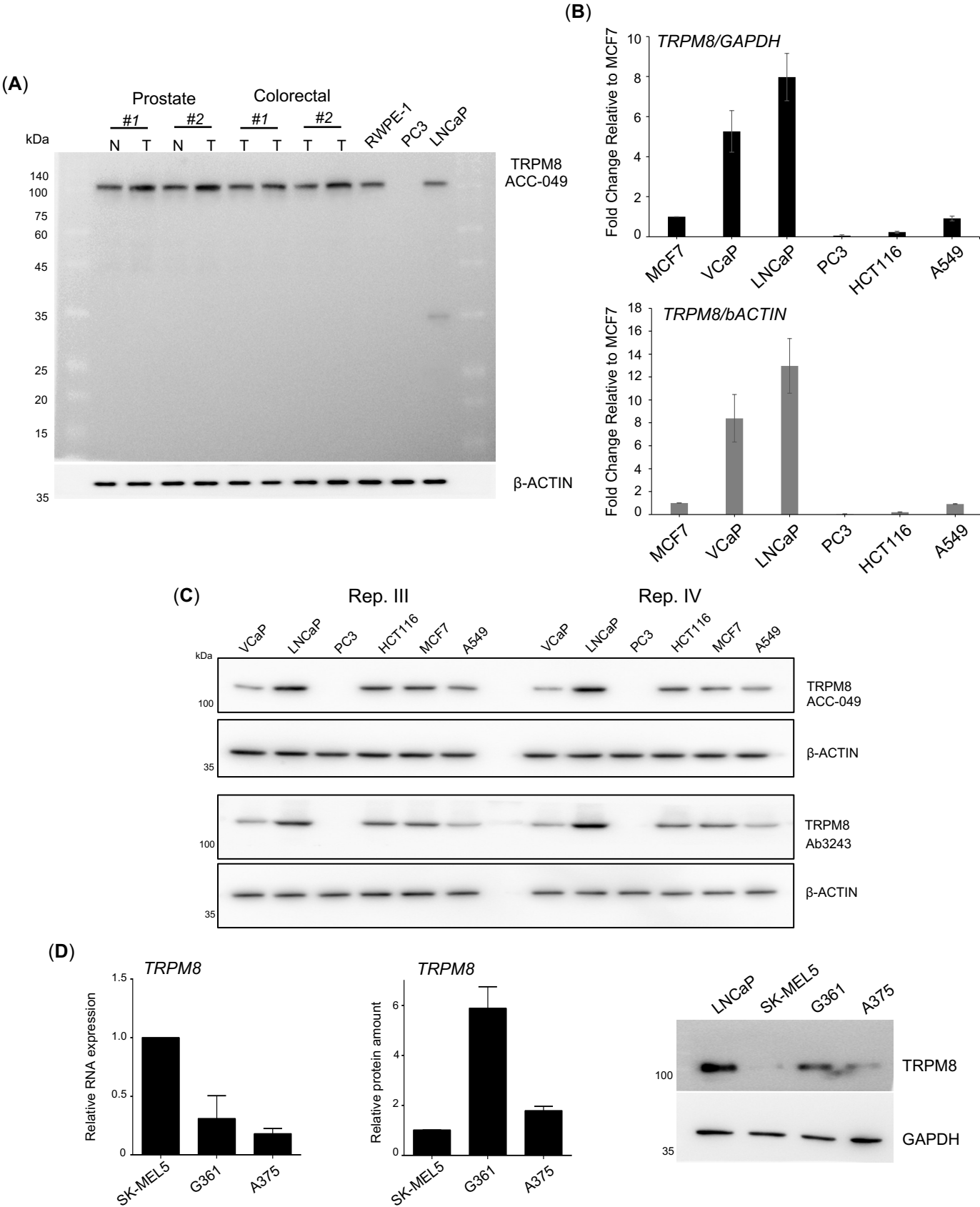

Supplement: Supplementary file 1 — Fig. S1. Comparable amounts of TRPM8 protein in prostate, colorectal, breast, and lung cancer cells. (A) Uncropped western blot relative to Fig. 1F. As previously described in Alaimo et al. (2020), lysates of LNCaP cells show both the full‐length (128 kDa, Plasma Membrane) and the shorter (35 kDa, Endoplasmic Reticulum) forms of TRPM8. (B) Amount of TRPM8 RNA in cancer cell lines relative to MCF7. Data are normalized using GAPDH (upper panel, n = 3) or bACTIN (lower panel, n = 2) expression as housekeeping genes. (C) Western blot replicas III and IV of TRPM8 in VCaP, LNCaP, PC3, HCT116, MCF7, A549 cell lines with the Alomone ACC‐049 (upper panel) and Abcam Ab3243 (lower panel) antibodies. β‐Actin is used as loading control. (D) TRPM8 RNA and protein quantification in melanoma cancer cell lines SK‐MEL5, G361, and A375 (n = 2). [file MOL2-19-2905-s007.pdf]

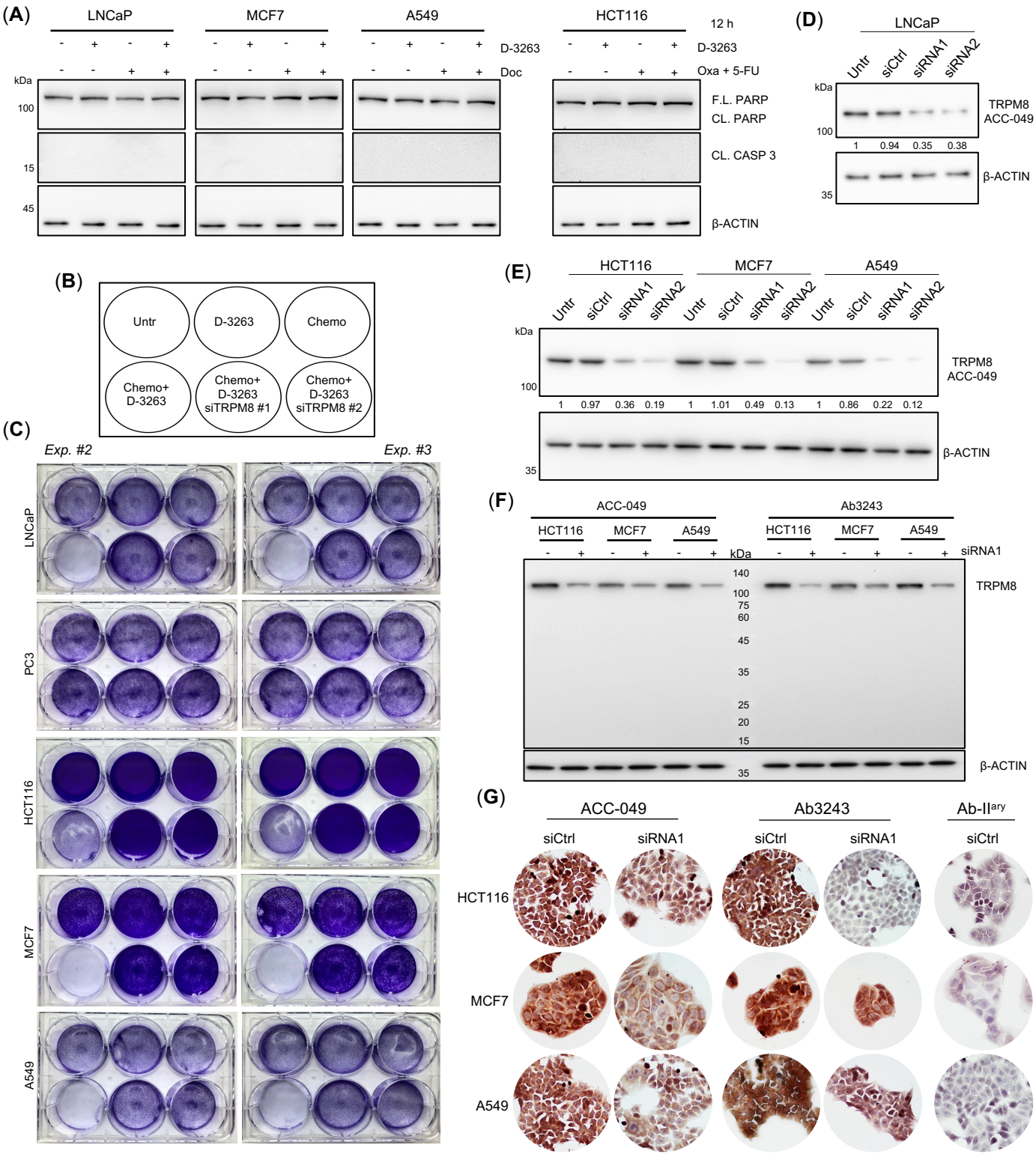

Supplement: Supplementary file 2 — Fig. S2. Activation of TRPM8 promotes chemotoxicity in cancer cells. (A) Western blot analysis of Caspase 3 and Parp cleavage in untreated or treated LNCaP, HCT116, MCF7, and A549 cell lines with the indicated drugs for 12 h. (B) Schematic representation of the experiments in C (chemotherapy = Docetaxel for LNCaP, MCF7, A549, and PC3; 5‐fluorouracile (5‐FU) + Oxaliplatin for HCT116. TRPM8 knock‐down = siTRPM8 #1 and siTRPM8 #2). (C) Crystal violet staining of LNCaP, HCT116, MCF7, A549, and PC3 cells untreated or treated for 24 h with the indicated drugs. (D, E) Western blotting of TRPM8 in LNCaP (D), HCT116, MCF7, and A549 (E) cell lines untransfected (Unt), transfected with control siRNA (siCtrl) or siRNAs targeting TRPM8 (siRNA1 and siRNA2). β‐Actin is used as loading control. Quantification is relative to the untreated (Untr) condition for each cell line. (F, G) Western blotting (F) and immunohistochemistry (G) of TRPM8 in HCT116, MCF7, and A549 cell lines transfected with control siRNA (−) or siRNA1 targeting TRPM8 (+). β‐Actin is used as loading control. Secondary antibody alone (Ab‐IIary) is used as negative control. [file MOL2-19-2905-s001.pdf]

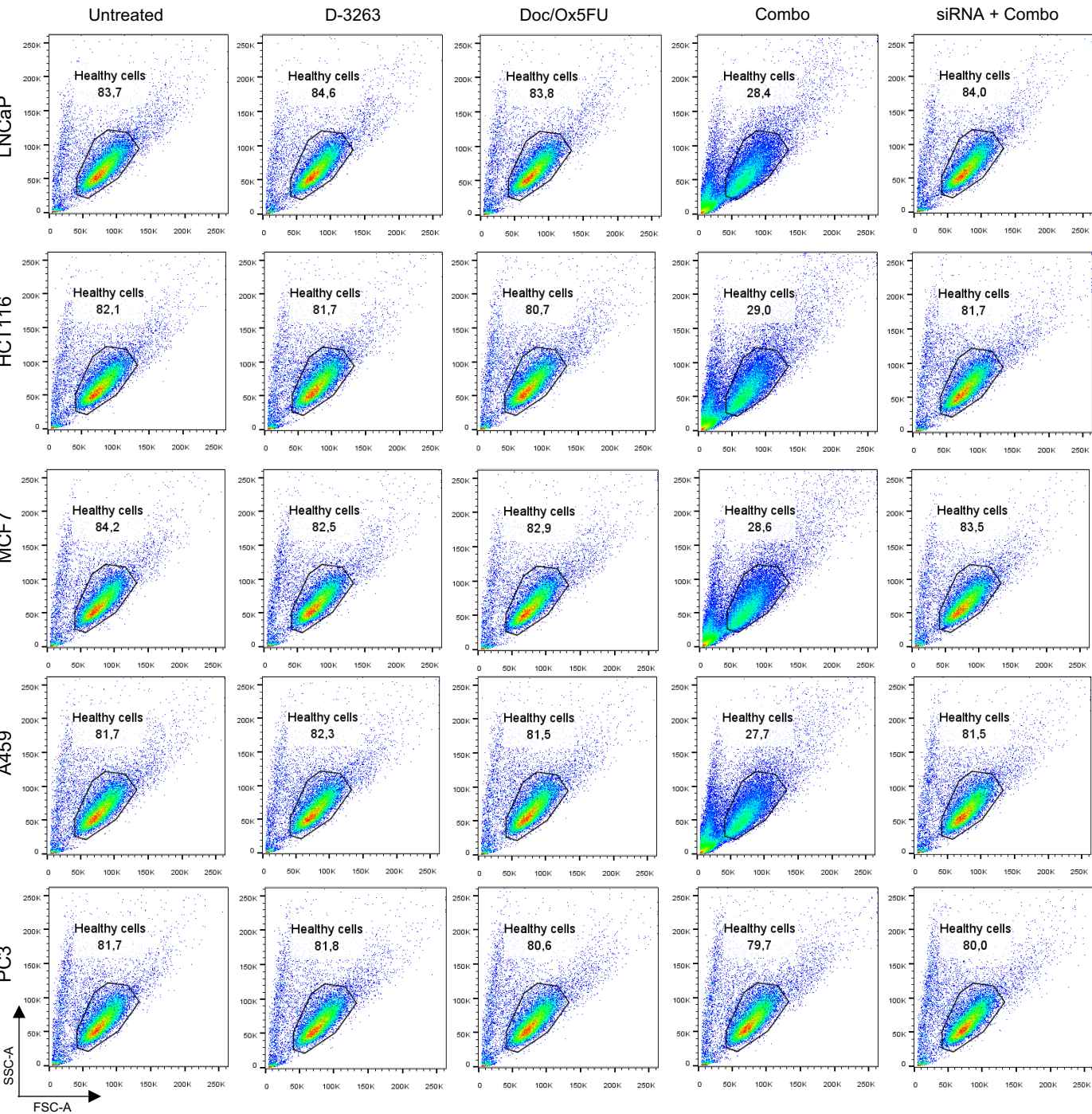

Supplement: Supplementary file 3 — Fig. S3. Combination of D‐3263 with chemotherapy induces apoptosis in TRPM8 positive cancer cells. Cell death rate by fluorescence‐activated cell sorting (FACS) with Annexin‐V‐FITC and propidium iodide (PI) staining of LNCaP, HCT116, MCF7, A549, and PC3 cells untreated or treated with the indicated drugs for 24 h (chemotherapy = Docetaxel for LNCaP, MCF7, A549, and PC3; 5‐fluorouracile (5‐FU) + Oxaliplatin for HCT116. TRPM8 knock‐down = siTRPM8 #2). [file MOL2-19-2905-s003.pdf]

(A)

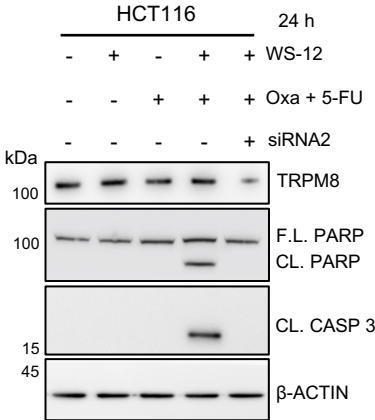

(B)

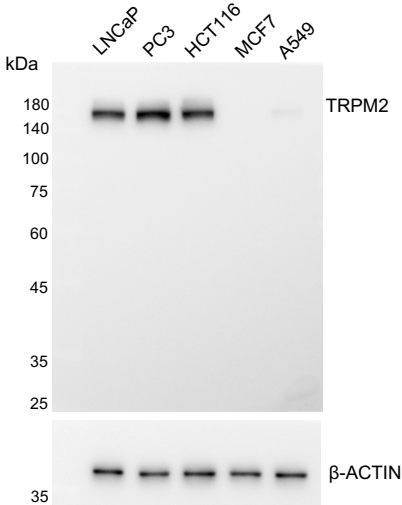

(C)

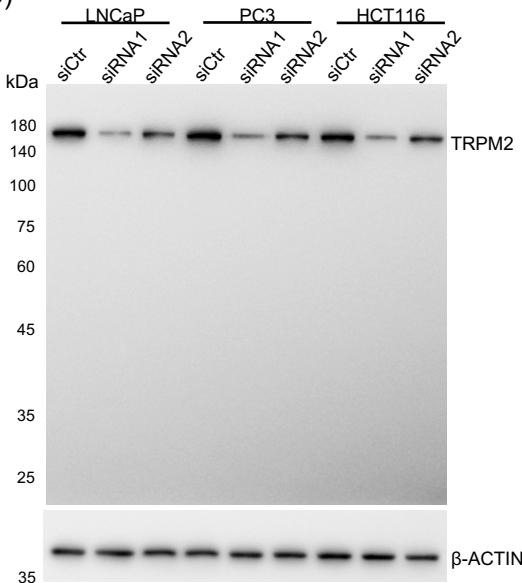

(D)

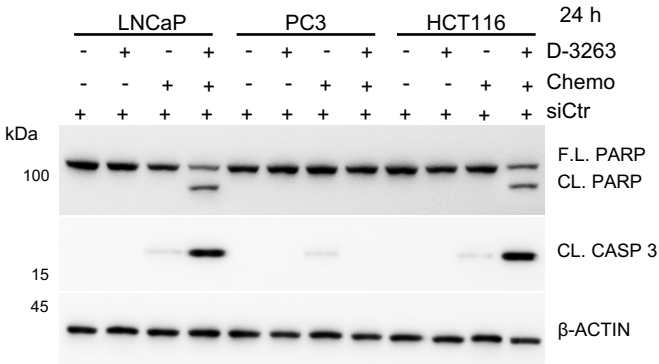

(E)

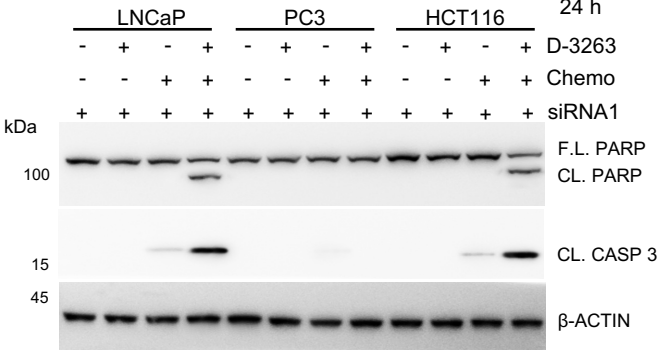

(F)

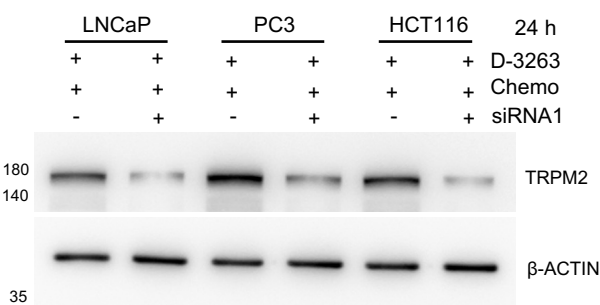

Supplement: Supplementary file 4 — Fig. S4. TRPM2 ion channel in cancer cell lines. (A) Western blotting analysis of TRPM8, PARP, and Caspase 3 in HCT116 cancer cell line treated with WS‐12 (1 M) and chemotherapy. (B) Western blotting analysis of TRPM2 expression in LNCaP, PC3, HCT116, MCF7 and A549 cancer cell lines. (C) Western blotting analysis showing TRPM2 knock‐down by specific siRNAs (siRNA1 and siRNA2) in LNCaP, PC3, HCT116 cancer cell lines. (D, E) Western blot analysis of Caspase 3 and PARP cleavage in LNCaP, HCT116, MCF7 cells transfected with control siRNA (siCtr) (D) or TRPM2 siRNA (siRNA1) (E) and untreated or treated with the indicated drugs for 24 h. (F) Western blotting analysis showing TRPM2 knock‐down by siRNA1 in LNCaP, PC3, HCT116 cancer cell lines treated with D‐3263 and chemotherapy in D and E. [file MOL2-19-2905-s002.pdf]
