# Supplementary material for: TRPM8 levels determine tumor vulnerability to channel agonists
Source: Mol Oncol. 2025 May 22;19(10):2905–20. doi: 10.1002/1878-0261.70049 (PMC12515718; doi:10.1002/1878-0261.70049)
Supplement: Supplementary file 5 — Fig. S5. TRPM8 ion channel expression in colorectal cancer specimens. (A) TRPM8 in a serial section to that shown in Fig. 4A of a homemade dedicated colorectal cancer tissue microarray (scale bar 100 μm; Ab3243). Representative images of CRC with different levels of TRPM8 staining and relative scores. Score 0: no expression; score 1: weak expression, score 2: moderate expression, score 3: high expression. (B) Distribution of TRPM8 immunostaining scores in colorectal cancer (CRC) samples with Alomone ACC‐049 and Abcam AB‐3243 antibodies, showing higher detection efficiency of ACC‐049 than Ab‐3243 at the same dilution, but consistent distribution of relative scores across samples. [file MOL2-19-2905-s006.pdf]

(**A**)

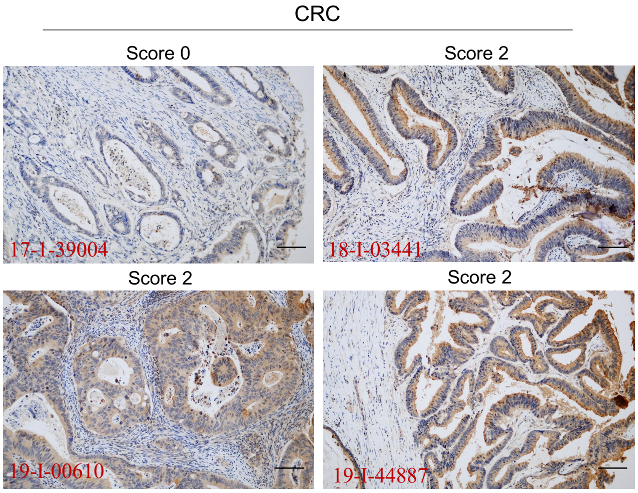

(**B**)

ID Sample	ACC-049	Ab-3243	ID Sample	ACC-049	Ab-3243
17-I-02639	*2*	*0*	19-I-07158	*2*	*1*
17-I-12188	*1*	*1*	19-I-21743	*1*	*1*
17-I-14606	*2*	*2*	19-I-35594	*1*	*2*
17-I-23341	*2*	*1*	19-I-44661	*2*	*3*
17-I-40214	*2*	*2*	19-I-03747	*2*	*1*
17-I-03093	*1*	*NA*	19-I-12264	*1*	*1*
17-I-14788	*2*	*1*	19-I-28348	*3*	*2*
17-I-23620	*2*	*2*	19-I-32119	*3*	*2*
17-I-28969	*2*	*1*	19-I-35731	*3*	*2*
17-I-36588	*2*	*1*	19-I-44887	*3*	*2*
17-I-41864	*1*	*2*	19-I-00610	*2*	*2*
17-I-09723	*3*	*3*	19-I-11878	*2*	*1*
17-I-18293	*1*	*1*	19-I-23977	*2*	*1*
17-I-22188	*3*	*2*	19-I-28211	*2*	*1*
17-I-28705	*2*	*1*	19-I-33233	*3*	*0*
17-I-39004	*1*	*0*	19-I-44998	*3*	*2*
17-I-41131	*2*	*2*	19-I-11857	*2*	*1*
17-I-05239	*2*	*2*	19-I-22237	*2*	*1*
17-I-25719	*2*	*1*	19-I-45003	*2*	*1*
17-I-39096	*3*	*2*	20-I-01396	*2*	*1*
18-I-03441	*2*	*2*	20-I-11094	*3*	*3*
18-I-09304	*3*	*2*	20-I-23206	*1*	*1*
18-I-26972	*3*	*2*	20-I-35630	*3*	*2*
18-I-33471	*3*	*2*	20-I-36035	*2*	*2*
18-I-43150	*2*	*2*	20-I-01878	*2*	*1*
18-I-00456	*2*	*2*	20-I-10475	*2*	*1*
18-I-12656	*3*	*2*	20-I-11210	*2*	*1*
18-I-15602	*2*	*2*	20-I-27620	*1*	*2*
18-I-28671	*3*	*2*	20-I-35634	*3*	*2*
18-I-40310	*1*	*1*	20-I-01326	*2*	*1*
18-I-00013	*2*	*1*	20-I-13258	*2*	*1*
18-I-05040	*3*	*2*	20-I-21866	*2*	*2*
18-I-09550	*2*	*1*	20-I-33281	*3*	*2*
18-I-33475	*3*	*2*	20-I-35463	*2*	*1*
18-I-39178	*2*	*2*	20-I-07353	*3*	*1*
18-I-42228	*3*	*1*	20-I-09556	*2*	*1*
18-I-12906	*2*	*2*	20-I-14408	*2*	*2*
18-I-31838	*3*	*2*	20-I-27968	*3*	*2*
18-I-29910	*3*	*NA*	20-I-34479	*3*	*2*
