# Supplementary material for: TRPM8 levels determine tumor vulnerability to channel agonists
Source: Mol Oncol. 2025 May 22;19(10):2905–20. doi: 10.1002/1878-0261.70049 (PMC12515718; doi:10.1002/1878-0261.70049)
Supplement: Supplementary file 6 — Table S1. Multi‐organ tissue microarray US Biomax, Inc. (MC2081a) and TRPM8 immunostaining score. [file MOL2-19-2905-s005.docx]

**Table S1**. Multi organ tissue microarray US Biomax, Inc. (MC2081a) and TRPM8 immunostaining score


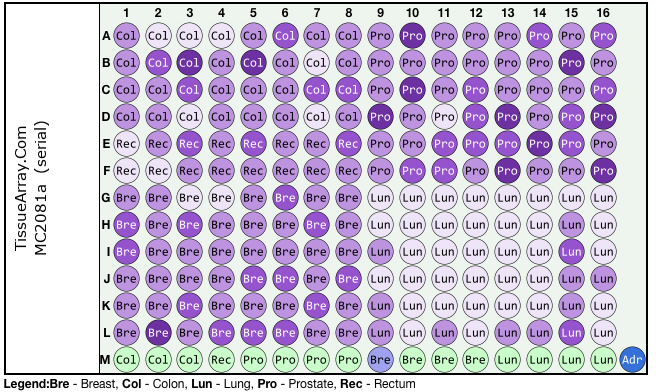


|  | 1 | 2 | 3 | 4 | 5 | 6 | 7 | 8 | 9 | 10 | 11 | 12 | 13 | 14 | 15 | 16 |  |
| --- | --- | --- | --- | --- | --- | --- | --- | --- | --- | --- | --- | --- | --- | --- | --- | --- | --- |
| A | 2 | 3 | 2 | 2 | 3 | 2 | 3 | 3 | 2 | 3 | 2 | 1 | 3 | 2 | 3 | 3 |  |
| B | 2 | 2 | 3 | 3 | 3 | 3 |  | 2 | 2 | 3 | 2 | 1 | 2 | 3 | 3 | 3 |  |
| C | 3 | 3 | 3 | 2 | 3 | 3 | 3 | 2 | 2 | 3 | 2 | 3 | 2 | 3 | 3 | 3 |  |
| D | 3 | 3 | 3 | 2 | 2 | 3 | 3 | 3 | 3 | 3 | 2 | 2 | 3 | 3 | 3 | 2 |  |
| E | 3 | 3 | 2 | 2 | 2 | 2 | 3 | 3 | 3 | 1 | 3 | 2 | 2 | 2 | 2 | 3 |  |
| F | 3 | 2 | 2 | 2 | 2 | 2 | 2 | 3 | 2 | 3 | 3 | 2 | 1 | 2 | 2 | 2 |  |
| G | 1 | 3 | 2 | 1 | 2 | 2 | 1 | 1 | 2 | 2 | 3 | 3 | 1 | 3 |  | 3 |  |
| H | 1 | 1 | 2 | 2 | 3 | 1 | 2 | 3 | 1 | 3 | 2 | 2 | 2 | 2 | 2 | 2 |  |
| I | 2 | 2 | 1 | 1 | 2 | 2 | 1 | 1 | 2 | 3 | 1 | 3 | 2 | 2 | 2 | 2 |  |
| J | 0 | 2 | 2 | 3 | 3 | 2 | 1 | 2 | 2 | 2 | 1 | 2 | 2 | 2 | 1 | 2 |  |
| K | 1 | 2 | 1 | 1 | 1 |  | 2 | 3 | 1 | 3 | 2 | 2 | 2 | 3 | 1 | 2 |  |
| L | 2 |  | 2 | 2 | 1 | 2 | 3 | 1 | 1 | 1 | 2 | 2 |  | 2 | 0 | 3 |  |
| M | 0 | 1 | 1 | 1 | 0 | 1 | 1 | 2 | 0 |  | 1 | 2 | 1 | 0 | 1 | 1 | 2 |

Row A-L = Tumour specimens

Row M = Normal tissue

Score 0 = Absent

Score 1 = Weak

Score 2 = Moderate

Score 3 = High
