# Supplementary material for: TRPM8 levels determine tumor vulnerability to channel agonists
Source: Mol Oncol. 2025 May 22;19(10):2905–20. doi: 10.1002/1878-0261.70049 (PMC12515718; doi:10.1002/1878-0261.70049)
Supplement: Supplementary file 8 — Table S3. Clinical description of Colorectal Cancer tissue microarray (TMA) and TRPM8 immunostaining score. [file MOL2-19-2905-s004.docx]

**Table S3.** Clinical description of Colorectal Cancer TMA and TRPM8 immunostaining score

Row 1: Colorectal tumours; Row 2: Adjacent normal tissues

Row 3: Colorectal tumours; Row 4: Adjacent normal tissues

Column F: Normal Spleen

| B **2017-1** | A | B | C | D | E | F |
| --- | --- | --- | --- | --- | --- | --- |
| 1 | 17-I-02639  Adenocarcinoma colic  T2; N0  G2;  Non-Mucinous  STAGE 1  (1.B4)  Score 2 | 17-I-12188  Adenocarcinoma partially differentiated  T2; N1b  G2;  Mucinous  STAGE 3a  (1.D6)  Score 1 | 17-I-14606  Adenocarcinoma  partially differentiated  T2; N0  G2;  Non-Mucinous  STAGE 1  (1.D3)  Score 2 | 17-I-23341  Adenocarcinoma  partially differentiated  T2; N1a  G2;  Non-Mucinous  STAGE 3a  (1.C1)  Score 2 | 17-I-40214  Adenocarcinoma ulcerate  T2; N0  G2;  Non-Mucinous  STAGE 1  (1.C4)  Score 2 | 21-A-00094  (A.10) |
| 2 | 17-I-02639  (1.B4)  Score 1 | 17-I-12188  (1.A2)  Score 1 | 17-I-14606  (1.D3)  Score 1 | 17-I-23341  (1.C1)  Score 1 | 17-I-40214  (1.C4)  Score 1 |  |
| 3 | 17-I-03093  Adenocarcinoma ulcerate  T3; N1  G2;  Non-Mucinous  STAGE 3b  (1.C3)  Score 1 | 17-I-14788  Adenocarcinoma partially differentiated  T3; N1  G2;  Non-Mucinous  STAGE 3b  (1.D1)  Score 2 | 17-I-23620  Adenocarcinoma partially differentiated ulcerate  T3; N1b  G2;  Non-Mucinous  STAGE 3b  (1.B3)  Score 2 | 17-I-28969  Adenocarcinoma partially differentiated  T3; N1a  G2;  Non-Mucinous  STAGE 3b  (2.A3)  Score 2 | 17-I-36588  Adenocarcinoma partially differentiated  ulcerate  T3; N1a  G2;  Non-Mucinous  STAGE 3b  (5.A6)  Score 2 |  |
| 4 | 17-I-03093  (1.B1)  Score 1 | 17-I-14788  (1.D1)  Score 1 | 17-I-23620  (1.A1)  Score 1 | 17-I-28969  (2.A3)  Score 1 | 17-I-36588  (5.A6)  Score 1 |  |

| B **2017-2** | A | B | C | D | E | F |
| --- | --- | --- | --- | --- | --- | --- |
| 1 | 17-I-41864  Adenocarcinoma invasive  T3; N1c, M1a  G2;  Non-Mucinous  STAGE 4a  (1.B2)  Score 1 | 17-I-09723  Adenocarcinoma partially differentiated  T4a; N2b, M1a  G3;  Mucinous  STAGE 4a  (1.D)  Score 3 | 17-I-18293  Adenocarcinoma ulcerate  T4a; N2a  G3;  Non-Mucinous  STAGE 3c  (1.C5)  Score 1 | 17-I-22188  Adenocarcinoma partially differentiated  T4a; N2a; M1c  G2;  Mucinous  STAGE 4c  (1.A2)  Score 3 | 17-I-28705  Adenocarcinoma partially differentiated  T4a; N0  G2;  Non-Mucinous  STAGE 2b  (1.B3)  Score 2 | 21-A-00094  (A.10) |
| 2 | 17-I-41864  (1.B2) | 17-I-09723  (1.B1) | 17-I-18293  (1.A1) | 17-I-22188  (1.C1) | 17-I-28705  (1.A1) |  |
| 3 | 17-I-39004  Adenocarcinoma partially differentiated  ulcerate  T4a; N2a, M1c  G2;  Non-Mucinous  STAGE 4c  (1.C3)  Score 1 | 17-I-41131  Adenocarcinoma partially differentiated  ulcerate  T4a; N0  G2;  Non-Mucinous  STAGE 2b  (1.B3)  Score 2 | 17-I-05239  Adenocarcinoma partially differentiated  T4b; N0  G2;  Non-Mucinous  STAGE 2c  (1.B4)  Score 2 | 17-I-25719  Adenocarcinoma partially differentiated  T4b; N0  G2;  Non-Mucinous  STAGE 2c  (1.I7)  Score 2 | 17-I-39096  Adenocarcinoma  ulcerate  T4b; N1a  G2;  Mucinous  STAGE 3c  (1.C1)  Score 3 |  |
| 4 | 17-I-39004  (1.C3)  Score 1 | 17-I-41131  (2.A1)  Score 1 | 17-I-05239  (1.B4)  Score 1 | 17-I-25719  (1.C1)  Score 1 | 17-I-39096  (1.B2)  Score 1 |  |

| B **2018-1** | A | B | C | D | E | F |
| --- | --- | --- | --- | --- | --- | --- |
| 1 | 18-I-03441  Adenocarcinoma partially differentiated  T2; N2a  G2;  Non-Mucinous  STAGE 3b  (1.C4)  Score 3 | 18-I-09304  Adenocarcinoma  T2; N0  G2;  Non-Mucinous  STAGE 1  (1.A1)  Score 3 | 18-I-26972  Adenocarcinoma partially differentiated  T2; N0  G2;  Mucinous  STAGE 1  (2.C1)  Score 3 | 18-I-33471  Adenocarcinoma partially differentiated  T2; N0  G2;  Non-Mucinous  STAGE 1  (2.B1)  Score 3 | 18-I-43150  Adenocarcinoma partially differentiated  T2; N1b  G2;  Non-Mucinous  STAGE 3a  (1.D6)  Score 2 | 21-A-00094  (A.10) |
| 2 | 18-I-03441  (1.B1)  Score 1 | 18-I-09304  (1.A1)  Score 1 | 18-I-26972  (2.C1)  Score 1 | 18-I-33471  (2.A1)  Score 1 | 18-I-43150  (1.D6)  Score 1 |  |
| 3 | 18-I-00456  Adenocarcinoma partially differentiated  T3; N1c  G3;  Non-Mucinous  STAGE 3b  (1.C1)  Score 2 | 18-I-12656  Adenocarcinoma partially differentiated  T3; N2  G2;  Non-Mucinous  STAGE 3b  (1.A1)  Score 3 | 18-I-15602  Adenocarcinoma partially differentiated  T3; N0, M1a  G2;  Non-Mucinous  STAGE 4a  (1.B2)  Score 2 |  | 18-I-28671  Adenocarcinoma poorly differentiated  T3; N2b  G2;  Non-Mucinous  STAGE 3c  (1.C1)  Score 3 |  |
| 4 | 18-I-00456  (1.D2)  Score 1 | 18-I-12656  (1.A1)  Score 1 | 18-I-15602  (1.B2)  Score 1 | 18-I-26141  (1.B4)  Score 1 | 18-I-28671  (1.C1)  Score 1 |  |

| B **2018-2** | A | B | C | D | E | F |
| --- | --- | --- | --- | --- | --- | --- |
| 1 | 18-I-40310  Adenocarcinoma partially differentiated  T3; N2a; M1a  G3;  Non-Mucinous  STAGE 4a  (3.C3)  Score 1 | 18-I-00013  Adenocarcinoma  ulcerate  T4a; N0; M1c  G3;  Non-Mucinous  STAGE 4c  (1.F4)  Score 2 | 18-I-05040  Adenocarcinoma partially differentiated  T4a; N2  G2;  Non-Mucinous  STAGE 3c  (3.B8)  Score 3 | 18-I-09550  Adenocarcinoma  ulcerate  T4a; N0  G2;  Non-Mucinous  STAGE 2b  (1.B1)  Score 2 | 18-I-33475  Adenocarcinoma  T4a; N2b  G3;  Non-Mucinous  STAGE 3c  (1.C5)  Score 3 | 21-A-00094  (A.10) |
| 2 | 18-I-40310  (3.C3)  Score 1 | 18-I-00013  (3.A6)  Score 1 | 18-I-05040  (3.B8)  Score 1 | 18-I-09550  (1.E1)  Score 1 | 18-I-33475  (1.C5)  Score 1 |  |
| 3 | 18-I-39178  Adenocarcinoma partially differentiated  ulcerate  T4a; N0  G2;  Non-Mucinous  STAGE 4a  (1.A3)  Score 2 | 18-I-42228  Adenocarcinoma partially differentiated  T4a; N1b; M1a  G2;  Non-Mucinous  STAGE 4a  (3.A2)  Score 3 | 18-I-12906  Adenocarcinoma partially differentiated  T4b; N1b  G2;  Non-Mucinous  STAGE 3c  (1.A4)  Score 2 | 18-I-31838  Adenocarcinoma partially differentiated  T4b; N1b  G2;  Non-Mucinous  STAGE 3c  (1.C1)  Score 3 | 18-I-29910  Adenocarcinoma partially differentiated  T4b; N2b; M1c  G3;  Non-Mucinous  STAGE 4c  (1.H1)  Score 3 |  |
| 4 | 18-I-39178  (1.A3)  Score 1 | 18-I-42228  (2.A1)  Score 1 | 18-I-12906  (1.A4)  Score 1 | 18-I-31838  (1.B1)  Score 1 | 18-I-29910  (1.H1)  Score 1 |  |

| **B 2019-1** | A | B | C | D | E | F |
| --- | --- | --- | --- | --- | --- | --- |
| 1 | 19-I-07158  Adenoma  colic  T2; N0  G2;  Non-Mucinous  STAGE 1  (1.A2)  Score 2 |  | 19-I-21743  Adenocarcinoma partially differentiated  T2; N0  G2;  Mucinous  STAGE 1  (1.A3)  Score 1 | 19-I-35594  Adenocarcinoma  T2; N0  G2;  Mucinous  STAGE 1  (1.B1)  Score 1 | 19-I-44661  Adenocarcinoma partially differentiated  T2; N0  G2;  Non-Mucinous  STAGE 1  (1.A4)  Score 2 | 21-A-00094  (A.10) |
| 2 | 19-I-07158  (1.A3)  Score 1 | 19-I-14343  (1.B2)  Score 1 | 19-I-21743  (1.A3)  Score 1 | 19-I-35594  (1.B1)  Score 1 | 19-I-44661  (1.A4)  Score 1 |  |
| 3 | 19-I-03747  Adenocarcinoma poorly differentiated  T3; N2a  G2;  Non-Mucinous  STAGE 3b  (1.C1)  Score 2 | 19-I-12264  Adenocarcinoma poorly differentiated  T3; N2a  G2;  Non-Mucinous  STAGE 3b  (1.C1)  Score 1 | 19-I-28348  Adenocarcinoma partially differentiated  T3; N1a  G2;  Non-Mucinous  STAGE 3b  (1.D4)  Score 3 | 19-I-32119  Adenocarcinoma  T3; N0  G3;  Non-Mucinous  STAGE 2a  (1.A4)  Score 3 | 19-I-35731  Adenocarcinoma partially differentiated  T3; N1a  G2;  Non-Mucinous  STAGE 3b  (1.C1)  Score 3 |  |
| 4 | 19-I-03747  (1.B1)  Score 1 | 19-I-12264  (1.C1)  Score 1 | 19-I-28348  (1.D4)  Score 1 | 19-I-32119  (1.A4)  Score 1 | 19-I-35731  (1.C1)  Score 1 |  |

| B **2019-2** | A | B | C | D | E | F |
| --- | --- | --- | --- | --- | --- | --- |
| 1 | 19-I-44887  Adenocarcinoma well  differentiated  T3; N0  G2;  Mucinous  STAGE 2a  (1.C2)  Score 3 | 19-I-00610  Adenocarcinoma partially differentiated  T4a; N1b; M1a  G2;  Non-Mucinous  STAGE 4a  (4.B2)  Score 2 | 19-I-11878  Adenocarcinoma  T4a; N2b  G2;  Non-Mucinous  STAGE 3c  (3.C6)  Score 2 | 19-I-23977  Adenocarcinoma  T4a; N1c  G2;  Non-Mucinous  STAGE 3b  (1.B1)  Score 2 | 19-I-28211  Adenocarcinoma  T4a; N1b  G2;  Non-Mucinous  STAGE 3b  (1.C1)  Score 2 | 21-A-00094  (A.10) |
| 2 | 19-I-44887  (1.C2)  Score 1 | 19-I-00610  (4.B2)  Score 1 | 19-I-11878  (3.C6)  Score 1 | 19-I-23977  (1.B1)  Score 1 | 19-I-28211  (1.C1)  Score 1 |  |
| 3 | 19-I-33233  Adenocarcinoma partially differentiated  ulcerate  T4a; N0  G2;  Mucinous  STAGE 2c  (1.B5)  Score 3 | 19-I-44998  Adenocarcinoma  T4a; N0  G3;  Non-Mucinous  STAGE 2c  (1.B2)  Score 3 | 19-I-11857  Adenocarcinoma colic  T4b; N2b  G2;  Non-Mucinous  STAGE 3c  (2.D2)  Score 2 | 19-I-22237  Adenocarcinoma partially differentiated  T4b; N1b  G2;  Non-Mucinous  STAGE 3c  (1.B1)  Score 2 | 19-I-45003  Adenocarcinoma  T4b; N1c  G2;  Non-Mucinous  STAGE 3c  (1.A2)  Score 2 |  |
| 4 | 19-I-33233  (1.B5)  Score 1 | 19-I-44998  (1.B2)  Score 1 | 19-I-11857  (2.D2)  Score 1 | 19-I-22237  (1.C1)  Score 1 | 19-I-45003  (1.D1)  Score 1 |  |

| B **2020-1** | A | B | C | D | E | F |
| --- | --- | --- | --- | --- | --- | --- |
| 1 | 20-I-01396  Adenocarcinoma colic  T2; N0  G2;  Non-Mucinous  STAGE 1  (1.B1)  Score 2 | 20-I-11094  Adenocarcinoma partially differentiated  T2; N0  G2;  Non-Mucinous  STAGE 1  (1.C4)  Score 3 | 20-I-23206  Adenocarcinoma colic  T2; N0  G2;  Non-Mucinous  STAGE 1  (1.A4)  Score 1 | 20-I-35630  Adenocarcinoma differentiated  T2; N1a  G2;  Non-Mucinous  STAGE 3a  (4.A3)  Score 3 | 20-I-36035  Adenocarcinoma invasive  T2; N0  G2;  Non-Mucinous  STAGE 1  (1.C4)  Score 2 | 21-A-00094  (A.10) |
| 2 | 20-I-01396  (1.B1)  Score 1 | 20-I-11094  (1.C4)  Score 1 | 20-I-23206  (1.A4)  Score 1 | 20-I-35630  (4.A3)  Score 1 | 20-I-36035  (1.A1)  Score 1 |  |
| 3 | 20-I-01878  Adenocarcinoma colic  T3; N2b  G2;  Non-Mucinous  STAGE 3c  (1.A3)  Score 2 | 20-I-10475  Adenocarcinoma partially differentiated  T3; N1a  G2;  Non-Mucinous  STAGE 3b  (1.C2)  Score 2 | 20-I-11210  Adenocarcinoma partially differentiated  T3; N2a  G2;  Non-Mucinous  STAGE 3b  (1.A1)  Score 2 | 20-I-27620  Adenocarcinoma  T3; N2a  G3;  Non-Mucinous  STAGE 3b  (1.A1)  Score 1 | 20-I-35634  Adenocarcinoma partially differentiated  T3; N0  G2;  Non-Mucinous  STAGE 2a  (1.A3)  Score 3 |  |
| 4 | 20-I-01878  (1.A3)  Score 1 | 20-I-10475  (1.C2)  Score 1 | 20-I-11210  (1.B1)  Score 1 | 20-I-27620  (2.A1)  Score 1 | 20-I-35634  (1.B1)  Score 1 |  |

| B **2020-2** | A | B | C | D | E | F |
| --- | --- | --- | --- | --- | --- | --- |
| 1 | 20-I-01326  Adenocarcinoma  T4a; N0  G1;  Non-Mucinous  STAGE 2b  (1.B1)  Score 2 | 20-I-13258  Adenocarcinoma invasive  T4a; N0  G2;  Non-Mucinous  STAGE 2b  (1.C1)  Score 2 | 20-I-21866  Adenocarcinoma poorly differentiated  T4a; N2a  G2;  Non-Mucinous  STAGE 3c  (1.D1)  Score 2 | 20-I-33281  Adenocarcinoma  T4a; N0  G2;  Non-Mucinous  STAGE 2b  (1.F3)  Score 3 | 20-I-35463  Adenocarcinoma  T4a; N1c  G3;  Non-Mucinous  STAGE 3b  (1.B5)  Score 2 | 21-A-00094  (A.10) |
| 2 | 20-I-01326  (1.D1)  Score 1 | 20-I-13258  (1.C1)  Score 1 | 20-I-21866  (1.D1)  Score 1 | 20-I-33281  (1.F3)  Score 1 | 20-I-35463  (1.B5)  Score 1 |  |
| 3 | 20-I-07353  Adenocarcinoma partially differentiated  T4b; N0  G3;  Non-Mucinous  STAGE 2c  (1.C2)  Score 3 | 20-I-09556  Adenocarcinoma  T4b; N1a  G2;  Non-Mucinous  STAGE 3c  (1.C1)  Score 2 | 20-I-14408  Adenocarcinoma partially differentiated  T4b; N1c; M1a  G2;  Non-Mucinous  STAGE 4a  (1.B2)  Score 2 | 20-I-27968  Adenocarcinoma poorly differentiated  T4b; N2b  G3;  Non-Mucinous  STAGE 3c  (1.J3)  Score 3 | 20-I-34479  Adenocarcinoma  T4b; N0  G3;  Non-Mucinous  STAGE 2c  (1.A2)  Score 3 |  |
| 4 | 20-I-07353  (1.C2)  Score 1 | 20-I-09556  (1.C1)  Score 1 | 20-I-14408  (1.B2)  Score 1 | 20-I-27968  (1.A1)  Score 1 | 20-I-34479  (1.D1)  Score 1 |  |
